# Sequencing the genome of *Marssonina brunnea* reveals fungus-poplar co-evolution

**DOI:** 10.1186/1471-2164-13-382

**Published:** 2012-08-09

**Authors:** Sheng Zhu, You-Zhi Cao, Cong Jiang, Bi-Yue Tan, Zhong Wang, Sisi Feng, Liang Zhang, Xiao-Hua Su, Brona Brejova, Tomas Vinar, Meng Xu, Ming-Xiu Wang, Shou-Gong Zhang, Min-Ren Huang, Rongling Wu, Yan Zhou

**Affiliations:** 1 Jiangsu Key Laboratory for Poplar Germplasm Enhancement and Variety Improvement, Nanjing Forestry University, Nanjing, China; 2 Center for Computational Biology, Beijing Forestry University, Beijing, China; 3 Shanghai-MOST Key Laboratory of Health and Disease Genomics, Chinese National Human Genome Center at Shanghai, Shanghai, China; 4 Research Institute of Forestry, Chinese Academy of Forestry, Beijing, China; 5 Faculty of Mathematics, Physics, and Informatics, Comenius University, Mlynska Dolina, Bratislava, 84248, Slovakia; 6 Department of Microbiology and Microbial Engineering, School of Life Sciences, Fudan University, Shanghai, China

**Keywords:** Marssonina leaf spot, Genome sequencing, Host-pathogen interaction, Poplar

## Abstract

**Background:**

The fungus *Marssonina brunnea* is a causal pathogen of Marssonina leaf spot that devastates poplar plantations by defoliating susceptible trees before normal fall leaf drop.

**Results:**

We sequence the genome of *M. brunnea* with a size of 52 Mb assembled into 89 scaffolds, representing the first sequenced *Dermateaceae* genome. By inoculating this fungus onto a poplar hybrid clone, we investigate how *M. brunnea* interacts and co-evolves with its host to colonize poplar leaves. While a handful of virulence genes in *M. brunnea*, mostly from the LysM family, are detected to up-regulate during infection, the poplar down-regulates its resistance genes, such as nucleotide binding site domains and leucine rich repeats, in response to infection. From 10,027 predicted proteins of *M. brunnea* in a comparison with those from poplar, we identify four poplar transferases that stimulate the host to resist *M. brunnea*. These transferas-encoding genes may have driven the co-evolution of *M. brunnea* and *Populus* during the process of infection and anti-infection.

**Conclusions:**

Our results from the draft sequence of the *M. brunnea* genome provide evidence for genome-genome interactions that play an important role in poplar-pathogen co-evolution. This knowledge could help to design effective strategies for controlling Marssonina leaf spot in poplar.

## Background

*Marssonina*, belonging to the family *Dermateaceae*, is an important fungus that causes Marssonina leaf spot, one of the most important and widespread foliage diseases, on all species of *Populus*[1-3]. Poplars infected with *Marssonina* are symptomized by small*,* scattered, circular to oval dead areas in the leaves, resulting in premature defoliation and, ultimately, weakening and dieback of the tree. Because of the continuing shrinkage of natural forests, fast-growing hybrid poplars have been increasingly planted worldwide in a short rotation intensive culture, aimed to maximize carbon sequestration and woody biomass production [4-6]. However, the infection of Marssonina leaf spot severely reduces the growth and productivity of hybrid poplars, leading to significant economic and ecological losses.

**Figure 1:**
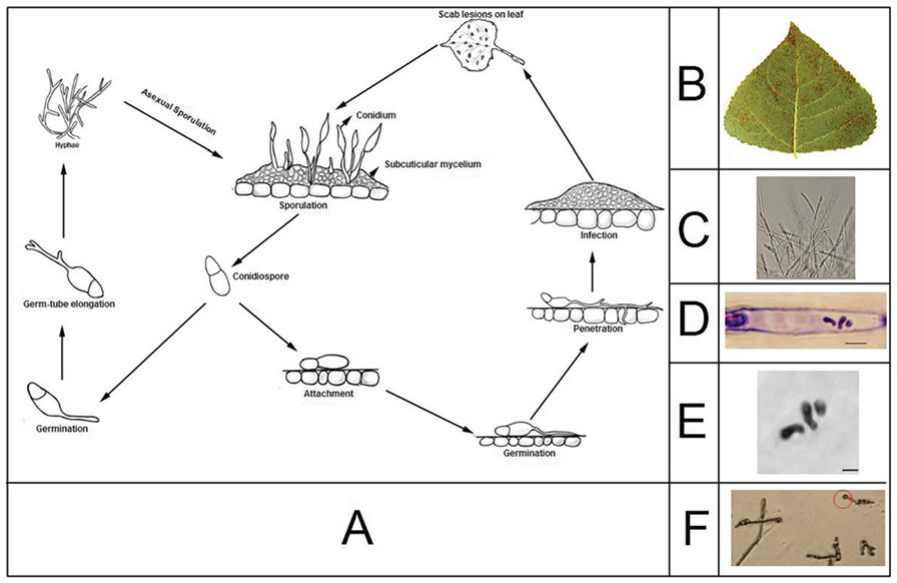
**Cytological karyotyping and life history of *M. brunnea. *(A**) Life cycle of *M. brunnea*. Conidia are asexual spores. The appressorium is a specialized infection structure. (**B**) The symptoms of Marssonina leaf spot include many small orange-brown spots on infected leaves. (**C**) The hyphae of *M .brunnea*. (**D**) Conventional light microscopy of asci from *M. brunnea*, scale bars = 2 *μm*. It is obvious that three bivalents are visible in this figure during Metaphase. (**E**) Mitotic metaphase chromosomes of *M. brunnea*, scale bars = 1 *μm*. Specimens prepared by the germ tube burst method were stained with Giemsa. This picture shows the spread of one nucleus containing full metaphase chromosome complements. (**F**) The appressoria of *M. brunnea* was marked as the red cycle.

Marssonina leaf spot is caused mainly by three species, *M. brunnea, M. castagnei*, and *M. populi*[2]. *M. brunnea* is a filamentous fungus with a relatively narrow host range. Figure 1 describes the life history of this fungus, its morphologies and cytological karyotyping. Both macroconidia and microconidia of *M. brunnea* are hyaline, but the former are unequally 2-celled and ovate or pear-shaped, whereas the latter is 1-celled and elliptical. In China, *M. brunnea* can be classified into two specialized forms, *M. brunnea f. sp. multigermtubi* and *M. brunnea f. sp. monogermtubi*[7]. Both forms infect leaves of poplar from *Aigeiros*, *Tacamahaca*, and *Leuce*, three of six sections of the *Populus* genus. At present, no fungicides are available for controlling Marssonina leaf spot and, thus, the most promising control is to plant poplar varieties resistant or tolerant to this disease. However, since the genetic mechanisms by which the fungus interacts with poplar to form Marssonina leaf spot are still elusive [8], our success to breed and select resistant poplar clones through marker-assisted and biotechnological approaches is very limited.

As a first step toward the selection of Marssonina-resistant poplars, we initiated a project for sequencing the genome of *M. brunnea*. In the past several years, more than 40 fungal genomes have been sequenced ( http://www.broadinstitute.org). However, most sequenced phytopathogenic fungi are those that colonize herbaceous plants, such as the rice blast pathogen *Magnaporthe grisea*[9], the corn smut pathogen *Ustilago maydis*[10], and the wheat head blight pathogen *Fusarium graminearum*[11]. Only a few studies have reported on the genome sequences of fungi parasitizing woody plants [12]. Apart from its economic value, *M. brunnea* can be used as a model system for studying pathogen-woody plant interactions because of its easy experimental manipulation, small genome size on three chromosomes and high genetic diversity [13,14].

In this study, we use a combination of Roche 454, ABI SOLiD, and Illumina/Solexa GA-II sequencing to sequence the genome of *M. brunnea*, in order to study the function of pathogenicity genes in this fungus. By comparing the *M. brunnea* genome with the genomes of two related fungi, *Botrytis cinerea* and *Sclerotinia sclerotiorum*[15], which have each evolved a different lifecycle, we further study the evolution and speciation of pathogenicity. In particular, by integrating it with the sequenced genome of the host poplar [16], the *M. brunnea* genome is used to identify protein-protein interactions between the pathogen and host. These findings could be translated into the development of effective and efficient strategies for controlling the pathogenesis of the disease and selecting resistant poplar clones.

## Results and Discussion

### The genome of *M. brunnea*

Using a combination of Roche 454, ABI SOLiD, and Illumina/Solexa GA-II sequencing, the genome of *M. brunnea* was sequenced to approximately 34-fold coverage, yielding 2,990 contigs and 155 scaffolds from a specialized form *M. brunnea f. sp. multigermtubi* (Additional file 1). The N50 scaffold length is 33,873 bp from the 4,532,414 Roche 454 reads with Newbler (v2.3). After gap filling, fewer contigs (2420) were assembled into 90 scaffolds with a larger N50 size, generating 52 Mb of assembled genome sequence (Table 1). We identified 28 s rRNA, 18 s rRNA and Internal Transcribed Spacer (ITS) using RNAmmer (v1.2) (Additional file 2).

**Table 1:** The main features of the genome

**Feature**	**Value**
Genomic Size	52 Mb
Coverage (fold)	Roche 454 (34x) + Solexa (97x) + SOLiD (66X)
GC content	42.71%
Protein-coding genes (> = 50 amino acids)	10,040
Average protein size (amino acids)	496
tRNA genes (genome)	119
tRNA genes (mitochondrion)	23
5S rRNA ^1^	30
28S rRNA^1^	1
18S rRNA ^1^	1

^1^ RNAmmer (v1.2, http://www.cbs.dtu.dk/services/RNAmmer/) was used to identity 5S, 28S, and 18S rDNA in *M. brunnea*.

Of the 192 gaps within the scaffolds that were filled using the Solexa contigs, three were coincident with the 27 gaps closed by primer walking, PCR, and sequencing. A preliminary finishing effort closed approximate 10% of the remaining genome gaps, some of which contained important regions, such as ITS and complete mitochondrial DNA. As an evaluation of the genome assembly scaffolds, 80.27% Solexa reads were mapped to the original 90 scaffolds as paired-end alignments using Bowtie (v0.12.7). Reads from Illumina/Solexa GA-II were de novo assembled into 53,924 contigs with a total of 51 Mb using Velvet (v1.0.02), of which 53,519 (99.25%) were aligned to the scaffolds.

Table 2 compares genome-wide proteins among the three closely related fungi, *B. cinerea*, *S. sclerotiorum* and *M. brunnea.* Of 14,522 proteins in *B. cinerea*, 10,699 (73.67%) were aligned to 9,928 proteins (68.37%) in *S. sclerotiorum*. Of 10,040 proteins in *M. brunnea,* 7,508 and 7528 were homologous to 8,154 of 9,928 proteins (82.13%) in *B. cinerea* and 8,907 of 10,699 proteins (83.25%) in *S. sclerotiorum*, respectively Table 3.

**Table 2:** Comparison of genome-scale proteins among three fungi

**Query**	**Subject**	
	**S. sclerotiorum**^**1**^	**B. cinerea**^**1**^
*S. sclerotiorum*	/	9,928 vs. 10,699
*M. brunnea*	7,652 vs. 8,380	7,648 vs. 9,095

^1^ The genome sequence of *B. cinerea* and *S. sclerotiorum* encode 16448 and 14522 proteins, respectively. Comparisons of genome-scale proteins were performed using BLASTP with an E-value < 1E-5.

**Table 3:** The distribution of repeats

**Type**	***M. brunnea***		***B. cinerea***		***S. sclerotiorum***	
	**Number**	**Length (bp)**	**Number**	**Length (bp)**	**Number**	**Length (bp)**
SINE^1^	0	0	0	0	0	0
DNA element	1,782	888,148	85	54,759	1,551	630,915
LINE^2^	502	583,811	0	0	1,712	671,898
LTR^3^	7,561	13,438,528	223	167,489	1,826	358,056
Low complexity	4,899	287,558	3,472	254,554	5,006	363,939
RC^4^	282	479,829	0	0	0	0
Satellite	1	41	0	0	1	64
Simple repeat	15,034	653,923	7,059	288,809	5,493	238,682
Unknown	9,122	5,519,980	1,099	197,624	1,572	243,540

^1^ SINE, short interspersed nuclear element.

^2^ LINE, long interspersed nuclear element.

^3^ LTR, long terminal repeat.

^4^ RC, rolling circle.

### Phylogenetic relationships

**Figure 2:**
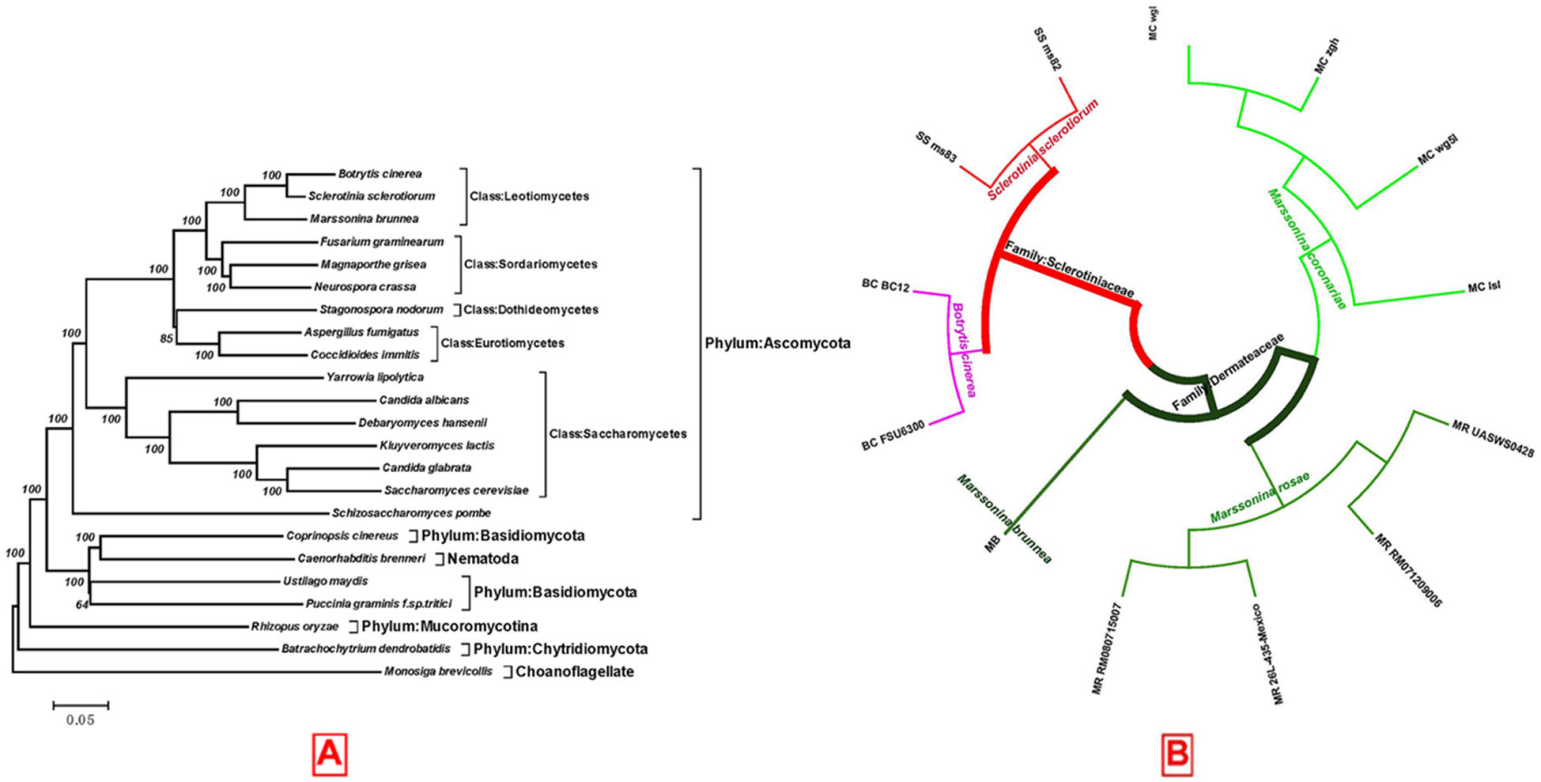
**Phylogenetic tree. (A)** Phylogenetic relationships among 21 fungi (including *M. brunnea*, *S. sclerotiorum*, *M. grisea*, *N. crassa*, *Fusarium graminearum*, *Aspergillus fumigatus, Coccidioides immitis, Stagonospora nodorum, Candida albicans, Debaryomyces hansenii, Candida glabrata, Saccharomyces cerevisiae, Kluyveromyces lactis, Yarrowia lipolytica, Schizosaccharomyces pombe, Coprinopsis cinereus, U. maydis, Puccinia graminis f. sp. tritici, Rhizopus oryzae, Batrachochytrium dendrobatidis,* and *Monosiga brevicollis*), *Caenorhabditis brenneri, and Marssonina coronariae*. To show the evolutionary relationships of the 23 species, a phylogenetic tree was constructed using the concatenated amino acid sequences with 1000 bootstraps. There were five main clusters, including Ascomycota fungi, Bssidiomycetes fungi and Nematoda, Mucoromycotina fungi, Chytridiomycosis fungi, and *Monosiga brevicollis* as outgroup. (**B**) The ITS sequences of *Marssonina coronariae* (MC, four strains), *Marssonina brunnea* (MB, *Marssonina brunnea f. sp. multigermtubi*), *Marssonina rosae* (MR, four strains), *Botrytis cinerea* (BC, two strains) and *Sclerotinia sclerotiorum* (SS, two strains) were used for constructing the phylogenetic tree. ITS sequences were aligned using ClustalW (v 2.1). A Neighbor-joining tree was built with 1000 bootstraps by MEGA (v4.0.2). The GenBank accession No. of the ITS sequences used for phylogenetic tree analysis are shown in Table S6.

Relatively little is known about the phylogenetic history of fungi because of a lack of their fossil records [17]. The concatenated amino acid sequences were used to construct a phylogenetic tree for 23 fungi [18,19] (Figure 2A), where *B. cinerea* and *S. sclerotiorum* are most closely related to *M. brunnea*, followed by *M. grisea*, *F. graminearum*, and *N. crassa* (Additional file 3), as supported by taxonomic positions among these fungi ( http://www.ncbi.nlm.nih.gov/Taxonomy/). However, pairwise comparisons indicated that *M. brunnea* only have 1,370 kb and 1,354 kb sequences similar to *B. cinere*a and *S. sclerotiorum*, respectively, suggesting that the former is evolutionarily distant to the latter two.

Due to a relatively rapid pace of change within the ITS1 and ITS2 sequences (Additional file 2), these regions can be suitably used to assess phylogenetic relationships among closely related taxa [20], including filamentous fungi at the species or genus level [21,22]. For example, ITS sequences were used for the phylogenetic analysis of genus *Lens* Mill [20] and species *Fusarium oxysporum*[23]. A neighbor-joining (NJ) phylogenetic tree was obtained using *B. cinerea* and *S. sclerotiorum* as outgroups (Figure 2B; Additontial file 4). The ITS sequence of *M. brunnea*, *M. rosae*, and *M. coronariae* were clustered as a group and were further subdivided into three sister subgroups. However, the ITS sequence of *M. brunnea* was also very similar to those of *B. cinerea* and *S. sclerotiorum*. By global alignment analysis with Needle (EMBOSS (v6.2.0) ( http://emboss.sourceforge.net/)), the ITS sequences of *Marssonina brunnea f. sp. multigermtubi* had a level of similarity of 59% with *B. cinerea* strain “FSU6300”, 68% with *B. cinerea* strain BC12, 70% with *S. sclerotiorum* strain “ms82”, and 72% with *S. sclerotiorum* strain “ms83”.

### Genome annotation

A total of 10,027 protein-coding genes were identified in the genome of *M. brunnea*. To measure the reliability of gene prediction, these predicted genes were compared by BLAST (BLASTP, E-value ≤ 1e-10) against CEGs (core eukaryotic genes) for orthologues [24,25]. The result from the comparative analysis showed 99% of orthologues (or 245 of 248 CEGs) found as full or partial genes and also indirectly suggested a relatively high reliability of gene prediction and completeness of the assembly. In addition, ~93% of the gene models (9340 predicted genes) were supported with unique RNA-seq reads.

There were 7,257 predicted proteins that were assigned potential functions by BLAST based on protein databases, including NR, UniProt, and KEGG. A total of 2,736 protein families containing 6,774 predicted proteins (Additional files 4 &5 ) were identified in *M. brunnea* using HMMER (v3.0) search against Pfam (v24). In addition, 288 (398 predicted proteins) and 61 (83 predicted proteins) protein families were identified by HMMER searching against Superfamily (v1.0) and TIGRFAM (v9.0), respectively.

Phi-base (pathogen-host interaction database) is a database that collects pathogenicity, virulence, and effector genes from fungi, oomycetes, and bacterial pathogens [26]. A total of 793 predicted genes shared homology to 622 of 924 genes in Phi-base (v3.2), when we used BLASTP with an E-value of <1E-10. Table S6 shows the number of proteins with more than 10 homologs from *M. brunnea*. By comparative functional analysis, the pathogenic genes were classified into six categories: (1) genes involved in recognizing the host and signal pathways, (2) genes affecting the biosynthesis of fungal cell wall and infection structure, (3) genes involved in degradation of the plant cuticle and cell wall, (4) genes involved in the pathogen protection mechanism during infection process (Additional files 6 &7), (5) genes whose roles are in fungal toxin biosynthesis (Additional file 8), and (6) fungal genes whose roles are in nutrient acquisition (Additional files 9 &10). We used a BLAST approach to infer the function of some of these genes, e.g., those related to sexual reproduction.

In the samples of *M. brunnea*, obtained from the eastern region of China, we did observe the asexual state but not the sexual state. Likewise, no sexual reproduction was found for *Marssonina* species in New Zealand Farm Forestry ( http://www.nzffa.org.nz/ farm-forestry-model/the-essentials/forest-health-pests-and-diseases/diseases/Marssonina/Poplar-anthracnose). To identity whether this fungus undergoes a sexual cycle, we used BLAST searching for orthologues of all genes related to sexual reproduction and meiosis (Additional file 11). Most of these sex-related genes were not found in *M. brunnea*. Some genes required for meiosis were present in *M. brunnea*, but they were involved in regulation as transcription factors or as supplementaries in syngenesis. For example, DMC1 related to meiosis were observed in *M. brunnea*, whereas those genes required for the formation of DMC1-containing nucleoprotein filaments were absent (Additional file 9) [27]. All these supported that *M. brunnea* might have no capacity to perform sexual reproduction.

### Transcriptome analysis

To compare gene-gene interactions between the pathogen and host, we used parallel massive sequencing of cDNA (RNA-seq) to estimate the relative expression levels of genes from *M. brunnea* and the host, poplar clone NL895 (*P. euramericana* CL“NL895”). Three cDNA libraries were constructed, including sample M6 of *M. brunnea* spores collected from potato dextrose agor, sample 895-M6 of clone NL895 leaves after 96 hours of infection by *M. brunnea*, and sample 895 of clone NL895 leaves with no infection.

RNA-seq reads from three different samples were aligned against the genome sequences of *M. brunnea* and *Populus* ( http://genome.jgi-psf.org/poplar/poplar.home.html) using TopHat (v1.1.4). Additional file 12 gives the mapping results of RNA-seq reads. Of the 10,040 predicted genes in *M .brunnea* and the 45,554 predicted genes in *Populus*, 9,340 (93%) and 31,794 (70%) were identified through RNA-seq, respectively, suggesting a relatively high coverage of the transcriptome.

**Figure 3:**
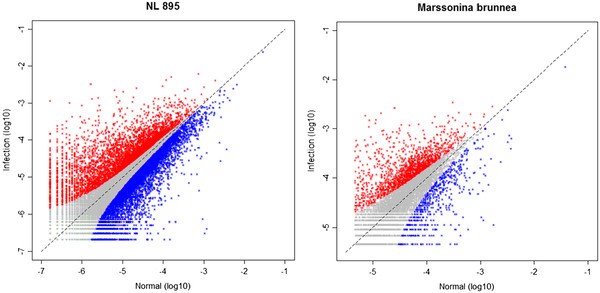
**Differential expression of the same genes in poplar leaves without infection (X-axis) and with infection by *M. brunnea *(Y-axis) (*p*** **< 0.001).** The amount of gene expression is described by a log10 ratio of the read number of the gene to total read number. Red, blue and gray dots represent up- regulation, down-regulation, and no differential expression, respectively. This figure thus shows the number of genes significantly up- or down-regulated for *M. brunnea* and *Populus* during early stages of infection.

The relative level of expression was calculated by using the amount of uniquely mapped reads for the annotated genes (Figure 3). There are 2,559 *M. brunnea* predicted genes that display different levels of expression from Sample M6 to Sample 895-M6 (*p* < 0.001), of which 1,898 are up-regulated. Most up-regulated genes come from the LysM family that functions effectors to suppress plant basal immunity during the colonization of plants [28-30], with 30 predicted genes from a total of 33 being significantly up-regulated (*p* < 0.001) (Additional files 13 &14). Other families that contain up-regulated genes (*p* < 0.001) are SNF2 family proteins, involved in such biological processes as transcription, DNA repair, chromatin-remodeling [31] and hyphae development [32], AMP-binding enzymes, playing a key role in degradation and synthesis of amino acids and lipids [33], and GDSL-like lipase/acylhydrolases family proteins [34] (Additional file 13). The numbers of up-regulated genes for these three families are 11, 11, and 10 from a total of 25, 26, and 15, respectively. No genes from the four families above were down-regulated, indicating that these gene families may play a pivotal role in the early stage of infection (96 hour post-inoculation).

We also compared differences of gene expression between Sample M6-895 and Sample 895. Of the 13,053 *Populus* genes that display such differences, 4,811 and 8,242 were up- and down-regulated, respectively. This comparison allows us to identify resistance proteins by which plants resist pathogenic attack [35]. A majority of plant resistant (R) genes contain nucleotide binding site domains (NBS) and leucine rich repeats (LRR), which are involved in the recognition of, and resistance to, pathogens [36,37]. Nine putative *Populus* R-genes were highly up-regulated at 96 hpi (*p* < 0.001) (Additional file 15), of which seven were the NBS-LRR type and two were the NBS and LRR (lrr1) types. Two *Populus* proteins, 815301 and 723016, similar to aminotransferases were significantly down-regulated (*p* < 0.001) for infected leaves, compared to uninfected ones. As aminotransferases regulating resistance to *P. cubensis* for melon [38], proteins At1 and At2, were significantly down-regulated in poplar (*p* < 0.001). These two proteins have a similar function to NSP-interacting kinases (NIKs) that mediate defense pathways in plants [39]. In *Arabidopsis*, NIK1 serves as a defense receptor that elicits the plant’s defense response [40].

Chitin widely exists in fungal cell walls and can be recognized by many LysM receptors in plants. The innate immunity of *Arabidopsis* was elicited when the LysM receptor CERK1 bounds to chitin [41,42]. There are 32 proteins containing the LysM domain in poplar, of which two (171810 and 233480) were significant down-regulated (*p* < 0.001) and shared homology with plant LysM receptor kinases, such as CERK1 in *Arabidopsis*. Perhaps it is possible that the putative LysM receptors in poplar were inhibited by LysM proteins in *M. brunnea* through competitive combination with fungal chitin.

All in all, most predict genes of *M. brunnea* and *Populus* could be detected in RNA-seq, some of which may play a crucial role in pathogen-host interactions, such as LysM motif-containing genes. The molecular mechanisms of the interactions between fungi and poplar have been studied through a complete description of the transcriptome of fungus-plant interactions.

### The co-evolution of *M. brunnea* and *Populus*

Like *Melampsora larici-populina* causing leaf rust of poplar [12], *M. brunnea* was an obligate plant pathogen to parasite poplar. There has been some evidence that obligate plant pathogens have co-evolved with their hosts expressed at the protein level [12]. Using the BLAST (BLASTP, E-value ≤ 1E-5) analysis, we found 8,093 predicted proteins of *M. brunnea* that are homologous to other eight fungal genomes, including *B. cinerea* (strain B05.10), *S. sclerotiorum* (strain 1980), *M. grisea* (strain 70–15), *F. graminearum* (strain PH-1), *U. maydis* (strain 521), *Schizosaccharomyces pombe* (strain 972 h-), *Saccharomyces cerevisiae* (strain RM11-1a), and *M. larici-populina* (strain 98AG31), as well as the *Populus* genome *P. trichocarpa* (poplar, v1.1), respectively. Of these proteins, 2,901 are homologous to each of the nine species, 265 are homologous to only one of the species, i.e., 96 to *B. cinerea*, 70 to *S. sclerotiorum*, 41 to *M. grisea*, 42 to *F. graminearum*, four to *U. maydis*, four to *S. pombe*, only one to *S. cerevisiae*, three to *M. larici-populina*, and four to *P. trichocarpa*.

The detection of more homologues to *B. cinerea* and *S. sclerotiorum* indirectly supported that *M. brunnea* is relatively more closely related to these two species than to the other species. One of the four homologues between *M. brunnea* and *P. trichocarpa* is M6_05232 that contains RNIG finger domain. The other three are glcG (M6_00711), 4-hydroxythreonine-4-phosphate dehydrogenase (M6_06189), and phosphomannomutase (M6_04436). Phosphomannomutase (PMM,EC5.4.2.8) pervading eukaryotes, such as *SEC53* in *S. cerevisiae*[43], *PMM1* in *Candida albicans*[44], and *At2g45790* in *Arabidopsis*[45], is a kind of phosphotransferases that participates in mannose metabolism. The genes encoding phosphomannomutase in *M. brunnea* has nonsignificant similarity to those in other species, which may be due to the high specificity of these genes in sequence and function generated in the co-evolution of *M. brunnea* and *Populus* as well their convergent evolution with a certain host genes. In addition, these genes decreased their expression remarkably 96 hours after the leaves of poplar were inoculated with *M. brunnea* (*p* < 0.001), but their *Populus* homologues did not produce any significant change in the level of expression. The change of expression of the PMM-encoding genes may arise from the alteration of how *M. brunnea* acquires energy after it invades poplar.

*M. brunnea* has three proteins that only have a significant sequence similarity to those in *M. larici-populina*, which are a secretory protein, a dynein heavy chain-like protein, and a glycosyltransferase 8 domain-containing (Pfam: F01501) protein. Glycosyltransferase 8 domain-containing gene was significantly up-regulated in the leaves of poplar infected after 96 hours (*p* < 0.001). In poplar fungal pathogens, such as *M. brunnea* and *M. larici-populina*, the glycosyltransferase 8 domain-containing protein may have played an important role in the assimilation of nutrients and the transportation of energy and carbohydrates from the poplar host. The four *Populus*-homologous genes, M6_05232, M6_00711, M6_06189, and M6_04436, derived from *M. brunnea* and *M. larici-populina* functions similarly in the infection of poplar leaves, suggesting that the environment where the two types of fungi live is an impetus for their genes to evolve into the same direction.

It is interesting to find that a putative galactokinase (EC 2.7.1.6) protein (M6_02750) of *M. brunnea* had a homologue only in *M. larici-populina* (jgi|Mellp1|115317) and *Populus* genomes (jgi|Poptr1_1|811685). Galactokinase is a phosphotransferase which has well been studied in many species, such as yeasts [46] and plants [47]. It is likely that this gene experiences co-evolution between the host and pathogen as well as between different obligate fungal pathogens that infect the same host, thus leading these three of species to produce a relatively high similarity in gene sequence. Also, the genes encoding galactokinase in *M. brunnea* and *M. larici-populina* produce convergent evolution with the homologues of their poplar host.

### Concluding Remarks

The genome of *Marssonina brunnea*, a woody plant pathogenic fungus that causes foliar disease in poplar, was sequenced and assembled with next-generation sequencing techniques, followed by a preliminary finishing effort that closed approximately 10% of the remaining gaps. After gap closure, the numbers of contigs and scaffolds decreased largely, accompanying an increasing size of N50 and the completion of a circular mtDNA. The genome sequence of *M. brunnea* reveals many important biological characteristics of the fungus, not only useful for studying the structure, organization and evolution of microbe genomes, but also shedding light on the molecular mechanisms of how pathogens and hosts interact and co-evolve.

A total of 28 LysM-containing proteins in *M. brunnea* were predicted as secreted proteins, which were significantly up-regulated during the process of infection. Interestingly, two LysM receptor-like kinases in *Populus* were significantly down-regulated after poplar is infected. These discoveries suggest that LysM proteins may play an important role in inhibiting the immunity system of poplar through competitive binding to chitin with the plant LysM receptor proteins.

We identified the genes that encode three types of transferases, i.e., phosphoglucomutase/ phosphomannomutase, glycosyltransferase 8 domain-containing protein, and galactokinase. These genes have driven *M. brunnea* and its *Populus* host to co-evolve, providing new insights into the genetic machineries of how obligate pathogenic fungi infect obligate hosts and how matter and energy flow and exchange between the pathogen and host. The completion of sequencing the *M. brunnea* genome opens a new resource for understanding the fundamental questions regarding pathogen-plant interactions, developing novel disease-control strategies and producing new disease-resistant varieties of tree crops.

## Experimental Procedures

### Strains

*Marssonina brunnea f. sp. multigermtubi* was obtained from the eastern region of China, including Shandong, Jiangsu, Henan, Shanxi, Jilin Provinces, and Beijing. It has been studied in our laboratory for approximately 30 years [2]. *M. brunnea f. sp. multigermtubi* (M6), which infects *Populus* species from Sections *Aigeiros* and *Tacamahaca*, was used as a sequenced reference strain.

### Sequencing

The sequencing of the genome of *M. brunnea f.sp multigermtubi* was performed at CHGC (Chinese National Human Genome Center at Shanghai). This yielded 4.5×10^6^ Roche 454 reads (4RUN) with an average length of 383 nt and a total size of 1.7 Gb. 4.7×10^7^ pairs of mate-paired reads (35 nt) with insert sizes of 5 kb were obtained from the SOLiD System. 2.1×10^7^ pairs of paired-end reads (120 nt) with insert sizes of 200 bp were obtained from the Illumina/Solexa GA-II. All PCR products for gap closure were sequenced using ABI 3730 xl DNA Analyzers.

Three RNA samples, i.e., M6 (*M. brunnea f. sp. multigermtubi*), 895 (leaves of poplar clone NL895 (*P. euramericana* CL“NL895”)) and 895-M6 (leaves of clone NL895 infected by *M. brunnea f. sp. multigermtubi*), were sequenced by the Illumina/Solexa GA-II. A dataset with 19.8 Gb or 73,228,774 reads with 113 nt reads length was produced.

### Assembly and gap closure

First, 4.5×10^6^ Roche 454 reads were assembled into 2,990 contigs by Newbler (v2.3). Then, 155 scaffolds were constructed using mate-paired information from SOLiD mate-paired reads and based on the algorithm of ConPath [48]. Using velvet (v1.0.02) [49], 2.1×10^7^ pairs of paired-end reads (120 nt) from Illumina/Solexa GA-II were de novo assembled into 53,924 Solexa contigs, with a total of 51 Mb. Based the information of order and direction of contigs within scaffolds, 192 gaps within scaffolds were closed using the 53,924 Solexa contigs. A total of 50 pairs of primers were designed to fill gaps between both adjacent contigs within scaffolds. A total of 27 gap sequences (with an average length of ~130 bp) were successfully filled, of which three gaps were coincident with that of 192 gaps using the Solexa contigs. After gap closure, the amount of initial contigs was reduced to just 2,420. Finally, a total of 90 scaffolds were reconstructed, with a total length of 52 Mb.

Next generation sequencing (NGS) short reads were mapped against the genome using Bowtie (v0.12.7) [50]. Solexa contigs were located to the genome sequences of *M. brunnea* using MEGABLAST ( http://www.ncbi.nlm.nih.gov/blast/megablast.shtml) with identity cut-off of 90%.

### Annotation

The gene prediction of *M. brunnea* was performed independently with a combination of three gene prediction program, including GeneMark (v2.3), Augustus (v2.3.1), and Exonhunter. The gene models were selected and manually curated by Argo Genome Browser (v 1.0.31, http://www.broadinstitute.org/annotation/argo/). The gene models were aligned using BLASTP against the protein sequence of *B. cinerea* and *S. sclerotiorum* ( http://www.broadinstitute.org/). The predicted proteins were identified using BLASTP against NR [51], KEGG [52], and UniProt [53].

The classification of protein families was done using HMMER (v3.0) against Pfam (v24), SupperFamily (v1.0), and TIGRFAM (v9.0). tRNA genes were detected using tRNAScan-SE (v1.23). Repetitive elements were screened using RepeatModeler (v 1.0.4) and RepeatMasker (v 3.2.9) (Additional files 16 &17). The analyses of putative transposon/retrotransposons were performed using Repbase (v 16.01). Secretory proteins were identified by a combination of SignalP (v 3.0) and TMHMM (v 2.0) ( http://www.cbs.dtu.dk/services/). The predicted secreted proteins in *M. brunnea* were aligned to the secretory proteins of six fungi (*U. maydis*, *M. grisea*, *B. cinerea*, *S. sclerotiorum*, *S. cerevisiae*, and *S. pombe*) from the Fungal Secretome Knowledge base [54] ( http://proteomics.ysu.edu/secretomes/fungi.php), using BLASTP with a cutoff E-value <1e-5 (Additional files 18 &19). Aligning genome-scale proteins against PHI-base (v3.2, http://www.phi-base.org/) [55] was performed by BLAST with an E-value of less than 1E-10 and to find putative gene involved in pathogenicity or virulence.

### Orthology and phyogenetic analysis

There are a total of 621 orthologous proteins which were obtained from *M. brunnea*, *B. cinerea* (B05.10), *and* 21 species which included 19 fungi, *Caenorhabditis brenneri and Marssonina coronariae* (Inparanoid (v7.0), http://inparanoid.sbc.su.se/cgi-bin/index.cgi). Multiple sequence alignments were done with ClustalW (v 2.1). A neighbor-joining (NJ) phylogenetic tree was constructed, based on concatenated protein sequences by MEGA (v4.0.2) with a bootstrap value of 1000.

To find potential synteny blocks between the *M. brunnea* genome and the genomes of *B. cinerea* and *S. sclerotiorum*, we used the BLAST analysis (BLASTN, with the alignment length of longer than 60 bp and over 75% identity) of the *M. brunnea* genome against the genomes of *B. cinerea* and *S. sclerotiorum*.

ITS (Internal transcribed spacer) sequences for *B. cinerea* and *S. sclerotiorum* were downloaded from the NCBI (Additional file 20). ITS sequences from *M. brunnea* were identified by ITS1 (tccgtaggtgaaccttgcgg) and ITS5 (ggaagtaaaagtcgtaacaagg). A Neighbor-joining (NJ) phylogenetic tree was constructed based on ITS sequences by MEGA (v4.0.2) with a bootstrap value of 1000.

### Digital transcriptome analysis

Poplar clone NL895, highly resistant to *M. brunnea f. sp. multigermtubi*, is one of the most important commercial planting clones in China. Cuttings of clone NL895 were cultured in the greenhouse at 22°C with a 12-hour photoperiod, until the cuttings were 0.5 ~ 1 m high and had 10 to 20 fully expanded leaves. Five or six fully expanded leaves were taken and placed on 2% water agar in sterile culture dishes with the abaxial surface uppermost. Conidia of *M. brunnea f. sp. multigermtubi* were suspended in sterile water. The spore suspensions were adjusted to 80,000 spores/ml and sprayed on the abaxial surface of the poplar leaves. Treated leaves were incubated in an illuminated incubator under 100% relative humidity (RH) at 22°C with a 12-hour photoperiod. Treated leaves were harvested at 4 days post-inoculation (dpi), then frozen quickly using liquid nitrogen, and stored at −70°C. RNA of the *M. brunnea f. sp. multigermtubi* conidia, uninfected leaves, and infected leaves were all extracted using Trizol reagent according to the manufacturer’s instructions (Invitrogen, Carlsbad, CA). Genomic DNA was removed by DNase I (TaKaRa, Japan).

RNA-seq reads were generated on an Illumina/Solexa GA-II. RNA-seq reads were mapped onto the genome of *M. brunnea* and *Populus trichocarpa* (v1.1, http://genome.jgi-psf.org/poplar/poplar.home.html), using a splice junction mapper named Tophat (v1.1.4, http://tophat.cbcb.umd.edu/). Differentially expressed genes were identified by determining the number of raw reads that uniquely mapped to genes, as a basis for determining significance by Fisher’s exact test and chi-square test.

### Accession numbers

The whole genome shotgun project has been submitted to GenBank/EMBL/DDBJ for *Marssonina brunnea .f.sp multigermtubi* (GeneBank accession No: AFXC00000000). ITS (GeneBank accession No: JN172909) and mitochondrial sequences (GenBank accession No: JN204424) of *M. brunnea .f.sp multigermtubi* are available.

## Competing interest

The authors declare that they have no competing interest.

## Authors’ contributions

SZ, YC, CJ, BT, ZW, SF performed data analysis, SZ, YC, CJ, BT, LZ, XS, BB, TV, MX carried out the experiments at different stages. MW, SZ, MH, RW, YZ conceived and designed the experiments. SZ, YC, CJ, BT, RW wrote the manuscript. All authors read and approved the final manuscript.

## Supplementary Material

Additional file 1 **Table S1.** Main features of *M. brunnea* genome assemblies. Click here for file

Additional file 2 **Figure S1.** The structure of ITS (internal transcribed spacer) DNA sequence. ITS1 was located between the SSU (small subunit) RNA and 5.8 s RNA, and ITS2 was located between the 5.8 s RNA and LSU (large subunit) RNA. Click here for file

Additional file 3 **Figure S2.** The taxonomic classification of three fungi including *M. brunnea*, *B. cinerea* and *S. sclerotiorum*. Click here for file

Additional file 4 **Figure S3.** The distribution of protein families in *M. brunnea*. Click here for file

Additional file 5 **Table S3.** Top 20 protein families in *M. brunnea* that are the most significantly different from those of other fungal genomes including *B. cinerea, S. sclerotiorum, M. grisea, and F. graminearum.* Click here for file

Additional file 6 **Figure S4.** Pathogen protection mechanism during infection. Fungi have mechanisms to avoid induction of the host immunity systems and alleviate the defense responses. The fungal plant pathogen *C. fulvum* gene ECP6 encodes a small, secreted protein, which sequesters chitin oligosaccharides to prevent eliciting host defense responses. Pathogens have two methods of coping with the toxicity and antifungal compound secreted by the host. One is efflux by the ABC1-encoded protein. The other is to produce enzymes to degrade them: *Gaeumannomyces graminis* secrets saponin-degrading enzymes AVENACINASE to detoxify the triterpenoid oat root saponin avenacin A-1. As the pathogens can secret some substances that contribute to infection that are also harmful to the pathogen itself, pathogen should encode methods of mitigating self-harm. *Fusarium sporotrichioides* can produce the trichothecene mycotoxin deoxynivalenol (DON) to inhibit protein synthesis of the host. The fungi have a gene called *TRI101* that encodes trichothecene 3-O acetyltransferase, which can reduce the damage to pathogen caused by trichothecene mycotoxin deoxynivalenol. Click here for file

Additional file 7 **Figure S5.** The domain structure for the gene *ABC3*. Click here for file

Additional file 8 **Figure S6.** Fungal toxin biosynthesis. Fungi produce toxins to destroy host cellular functions. They can be non-host specific or host specific. Fungi have many genes to control the biosynthesis, export, and regulation of the toxins. Cercosporin is a non-host specific toxin. A polyketide synthase gene, CTB1, plays a key role in cercosporin biosynthesis. CFP encodes a cercosporin Transporter exporting cercosporin, CZK3, which regulates cercosporin biosynthesis. Comparing to the non-host specific toxins, some toxins are active only toward hosts, i.e. host specific toxins, such as HC-toxin, AK-toxin, AM-toxin, and ACT-toxin. HTS1 encodes a multifunctional cyclic peptide synthetase involved in the biosynthesis of HC-toxin. Besides HTS1, ToxC and ToxF are also essential for toxin biosynthesis and pathogenicity. AKT1, which encodes a series of carboxyl-activating enzymes, and AKT2 are involved in the biosynthesis of the AK-toxin. The AMT gene is essential for the biosynthesis of the AM-toxin. ACTTS2 and ACTTS3 are essential genes for ACT-toxin biosynthesis. Click here for file

Additional file 9 **Text S1.** Additional Description. Click here for file

Additional file 10 **Table S4.** Six gene groups involved in pathogenesis. Click here for file

Additional file 11 **Table S5.** The genes associated with mating and meiosis. Click here for file

Additional file 12 **Table S6.** The number of RNA-seq reads mapped to the genome of *Populus* and *M. brunnea.* Click here for file

Additional file 13 **Table S7.** Protein families with more than 10 genes that were up-regulated in *M. brunnea*. Click here for file

Additional file 14 **Figure S7.** Multiple alignment of 28 putative proteins with highly similarity for *M. brunnea.* Multiple sequence alignment of the 28 putative proteins was performed using ClustalW. Click here for file

Additional file 15 **Table S8.** Resistance genes (R) with differential expression in *Populus*. Click here for file

Additional file 16 **Table S9.** The distribution of low complexity sequences for *M. brunnea, B. cinerea, and S. sclerotiorum*. Click here for file

Additional file 17 **Table S10.** The distribution of simple repeat sequences for *M. brunnea, B. cinerea, and S. sclerotiorum*. Click here for file

Additional file 18 **Table S11.** The number of putative secretory proteins among *U. maydis*, *M. grisea*, *B. cinerea*, *S. sclerotiorum*, and *M. brunnea.* Click here for file

Additional file 19 **Table S12.** The secretory protein families with more than five members *M. brunnea*. Click here for file

Additional file 20 **Table S2.** The GenBank accession no of ITS sequences used for phylogenetic tree analysis. Click here for file
