# Supplementary material for: Sequencing the genome of Marssonina brunnea reveals fungus-poplar co-evolution
Source: BMC Genomics. 2012 Aug 9;13:382. doi: 10.1186/1471-2164-13-382 (PMC3484023; doi:10.1186/1471-2164-13-382)
Supplement: Additional file 1 — Table S1. Main features of M. brunnea genome assemblies. [file 1471-2164-13-382-S1.doc]

Table S1 Main features of *M. brunnea* genome assemblies.

|  | Non-gap Closure | | Gap Closure | |
| --- | --- | --- | --- | --- |
|  | Contig | Scaffold | Contig | Scaffold |
| No. of contig/scaffold | 2990 | 155 | 2420 | 90 |
| N50 size of contig/scaffold | 33,873 | 1,359,333 | 39,335 | 1,599,552 |
| Mean length of contig/scaffold | 17,565 | 340,660 | 21,398 | 577,958 |
| Minimum length of contig/scaffold | 1002 | 1006 | 1002 | 1006 |
| Maximum length of contig/scaffold | 235,302 | 3,606,805 | 246,791 | 5,185,404 |
| Genome Size | 52.5Mbp | 52.8Mbp | 51.78Mbp | 52Mbp |
| GC-content (%) | 42.69% | 42.46% | 42.90% | 42.71% |

N50 is widely used for measuring the average length of assembled sequences in genome assembly and is defined as the largest length N for which the sum of the sequences that are more than or equal the length N are not less than half of the genome sizes.
