# Supplementary figures and images for: Sequencing the genome of Marssonina brunnea reveals fungus-poplar co-evolution
Source: BMC Genomics. 2012 Aug 9;13:382. doi: 10.1186/1471-2164-13-382 (PMC3484023; doi:10.1186/1471-2164-13-382)

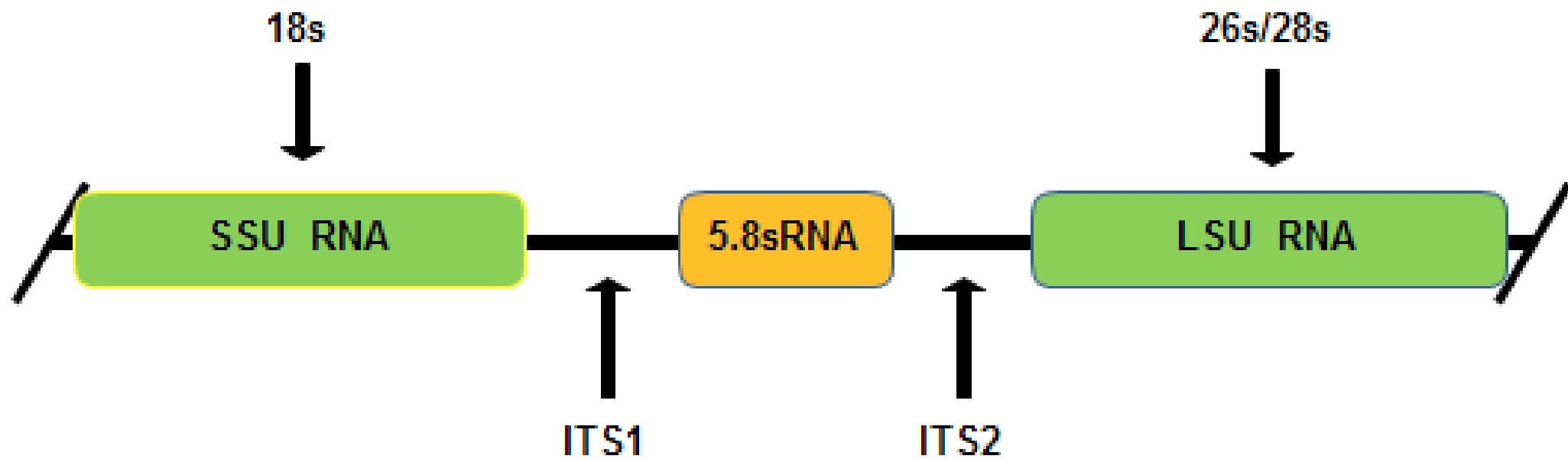

Supplement: Additional file 2 — Figure S1. The structure of ITS (internal transcribed spacer) DNA sequence. ITS1 was located between the SSU (small subunit) RNA and 5.8 s RNA, and ITS2 was located between the 5.8 s RNA and LSU (large subunit) RNA. [file 1471-2164-13-382-S2.pdf]

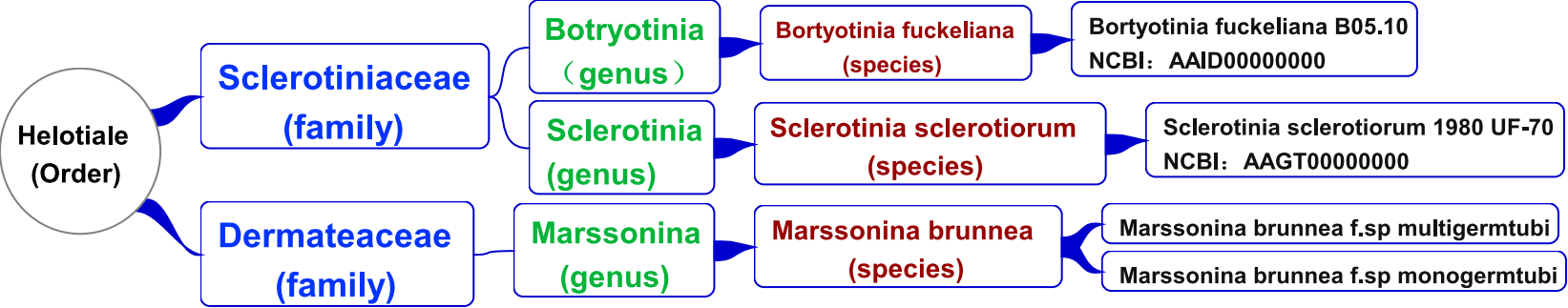

Supplement: Additional file 3 — Figure S2. The taxonomic classification of three fungi including M. brunnea, B. cinerea and S. sclerotiorum. [file 1471-2164-13-382-S3.pdf]

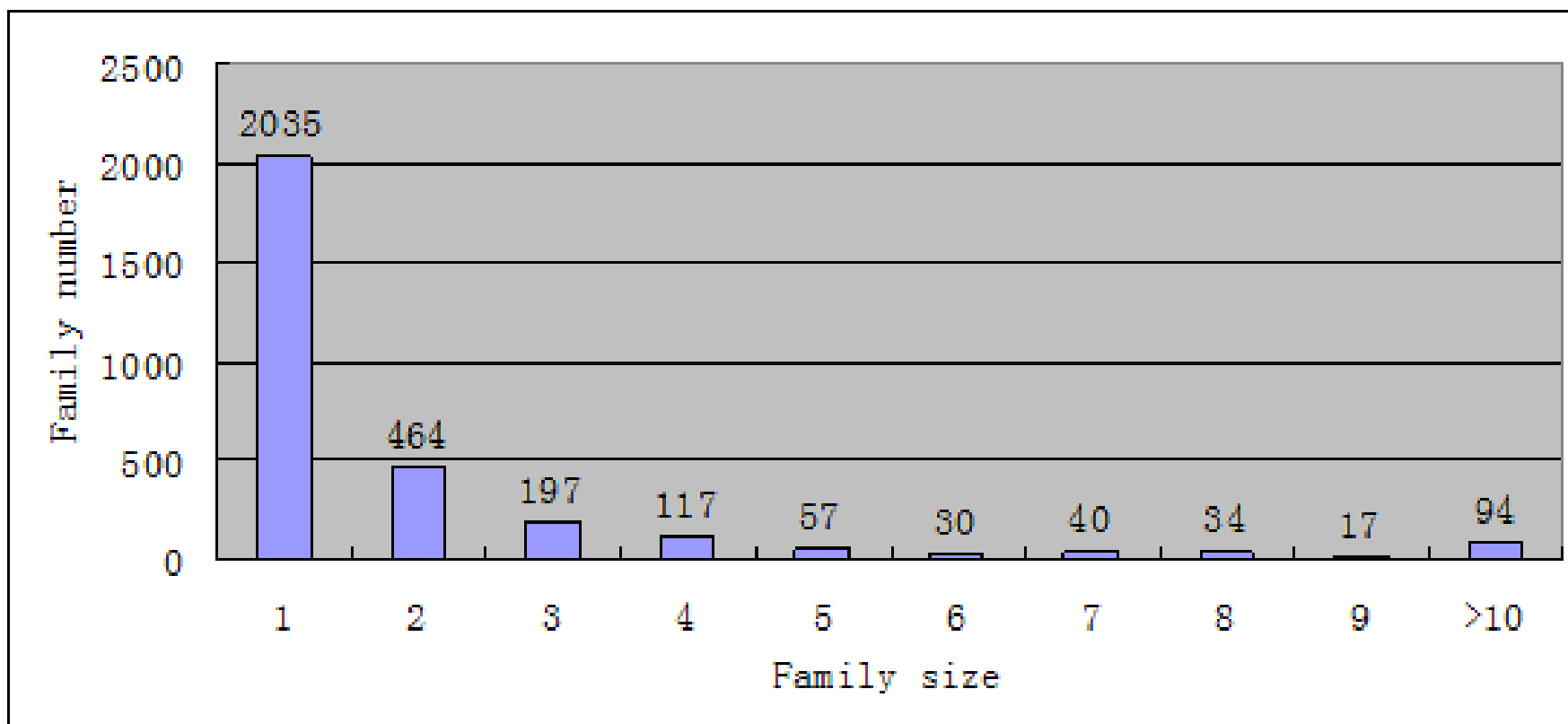

Supplement: Additional file 4 — Figure S3. The distribution of protein families in M. brunnea. [file 1471-2164-13-382-S4.pdf]

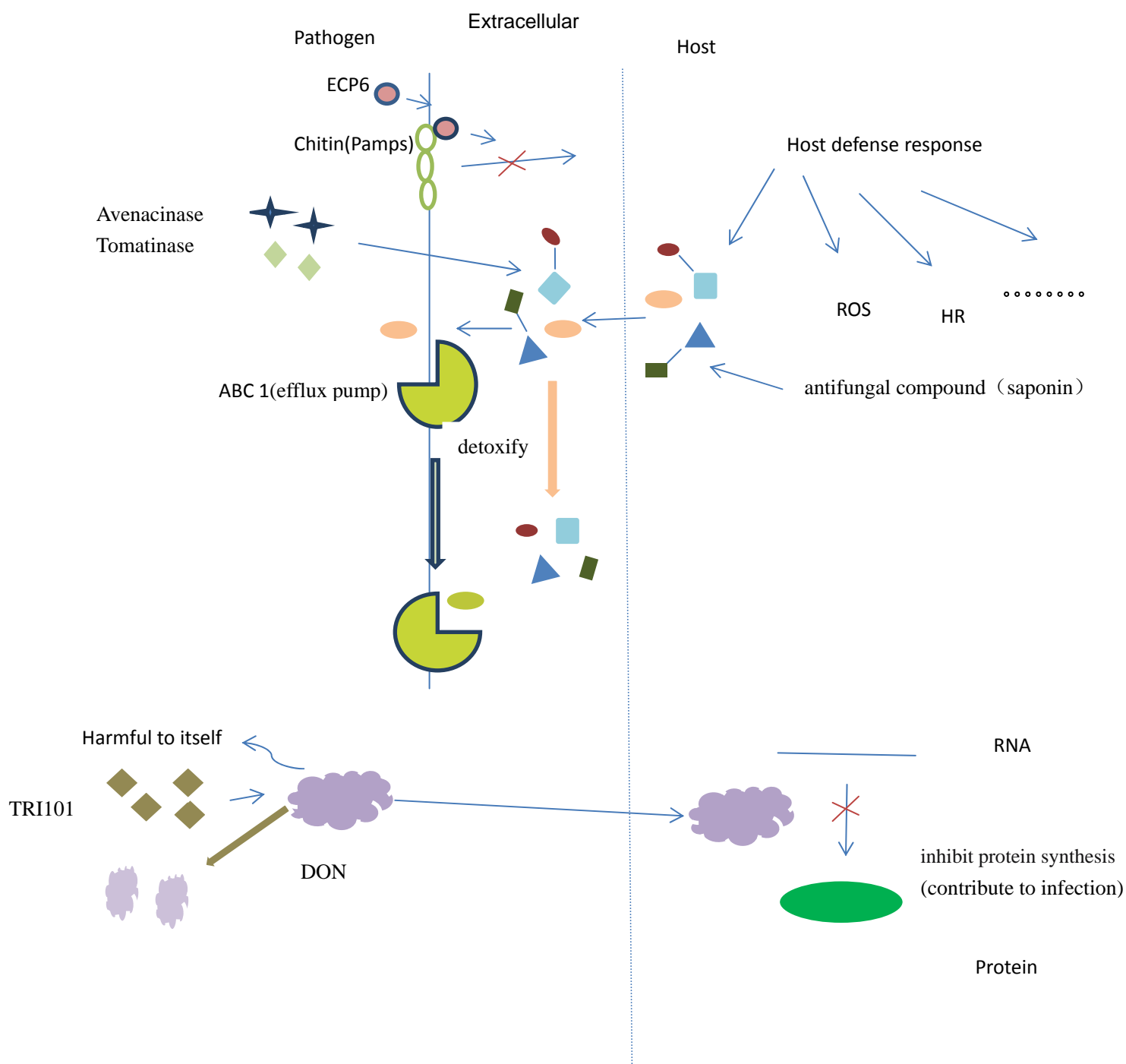

Supplement: Additional file 6 — Figure S4. Pathogen protection mechanism during infection. Fungi have mechanisms to avoid induction of the host immunity systems and alleviate the defense responses. The fungal plant pathogen C. fulvum gene ECP6 encodes a small, secreted protein, which sequesters chitin oligosaccharides to prevent eliciting host defense responses. Pathogens have two methods of coping with the toxicity and antifungal compound secreted by the host. One is efflux by the ABC1-encoded protein. The other is to produce enzymes to degrade them: Gaeumannomyces graminis secrets saponin-degrading enzymes AVENACINASE to detoxify the triterpenoid oat root saponin avenacin A-1. As the pathogens can secret some substances that contribute to infection that are also harmful to the pathogen itself, pathogen should encode methods of mitigating self-harm. Fusarium sporotrichioides can produce the trichothecene mycotoxin deoxynivalenol (DON) to inhibit protein synthesis of the host. The fungi have a gene called TRI101 that encodes trichothecene 3-O acetyltransferase, which can reduce the damage to pathogen caused by trichothecene mycotoxin deoxynivalenol. [file 1471-2164-13-382-S6.pdf]

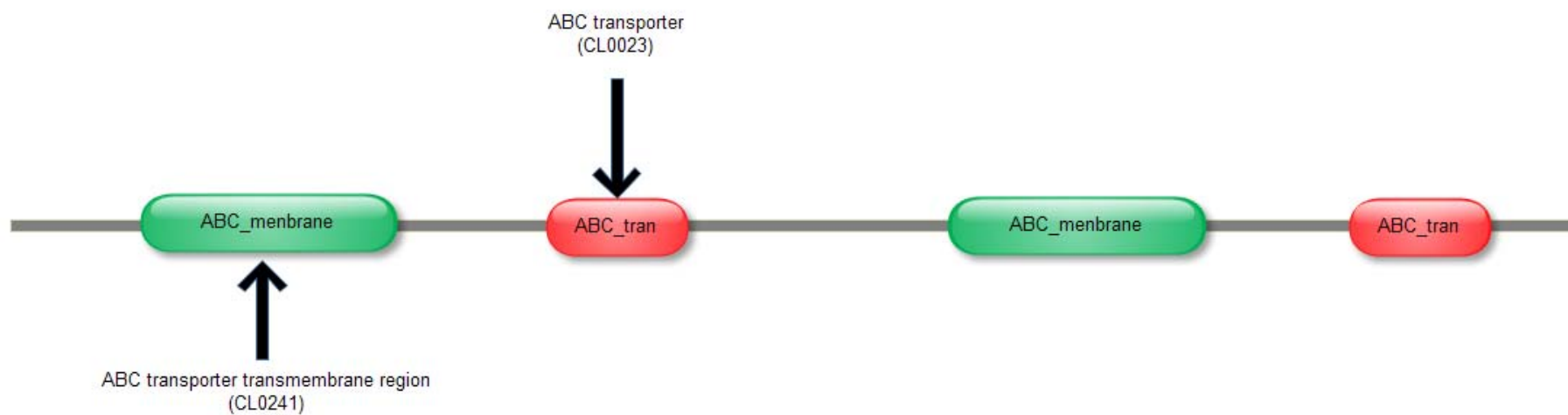

Supplement: Additional file 7 — Figure S5. The domain structure for the gene ABC3. [file 1471-2164-13-382-S7.pdf]

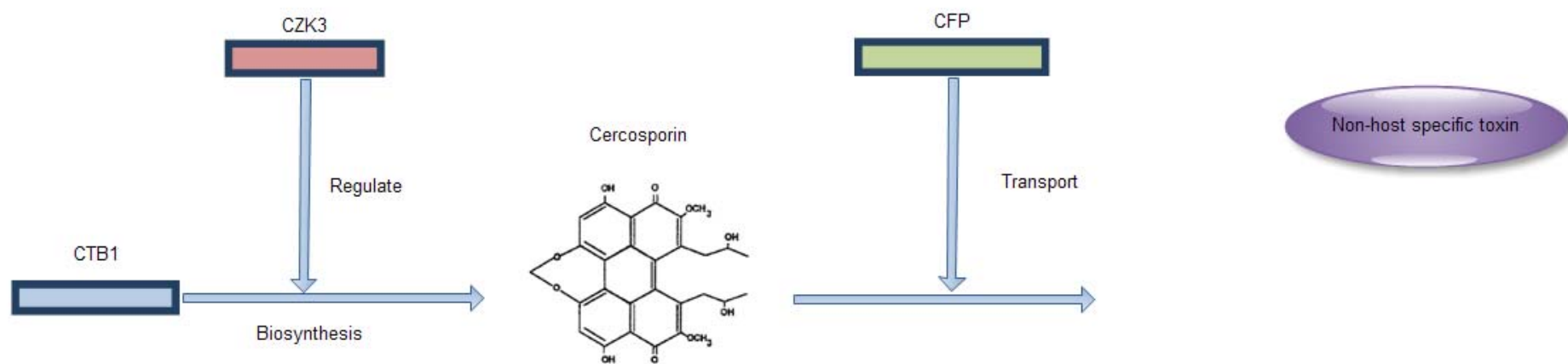

HTS1  
TOXC  
TOXF

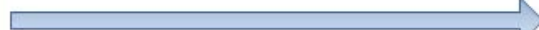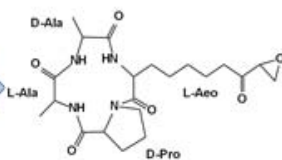

HC-toxin

AKT1  
AKT2

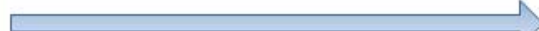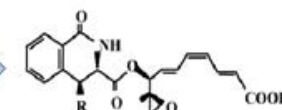

AK-toxin

AMT

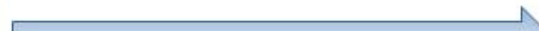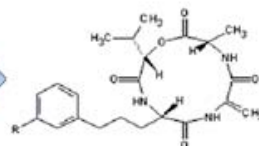

AM-toxin

ACTTS2  
ACTTS3

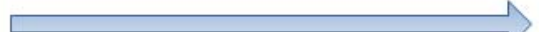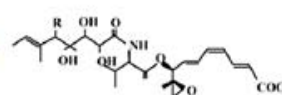

ACT-toxin

Host specific toxin

Supplement: Additional file 8 — Figure S6. Fungal toxin biosynthesis. Fungi produce toxins to destroy host cellular functions. They can be non-host specific or host specific. Fungi have many genes to control the biosynthesis, export, and regulation of the toxins. Cercosporin is a non-host specific toxin. A polyketide synthase gene, CTB1, plays a key role in cercosporin biosynthesis. CFP encodes a cercosporin Transporter exporting cercosporin, CZK3, which regulates cercosporin biosynthesis. Comparing to the non-host specific toxins, some toxins are active only toward hosts, i.e. host specific toxins, such as HC-toxin, AK-toxin, AM-toxin, and ACT-toxin. HTS1 encodes a multifunctional cyclic peptide synthetase involved in the biosynthesis of HC-toxin. Besides HTS1, ToxC and ToxF are also essential for toxin biosynthesis and pathogenicity. AKT1, which encodes a series of carboxyl-activating enzymes, and AKT2 are involved in the biosynthesis of the AK-toxin. The AMT gene is essential for the biosynthesis of the AM-toxin. ACTTS2 and ACTTS3 are essential genes for ACT-toxin biosynthesis. [file 1471-2164-13-382-S8.pdf]
