# Supplementary material for: Sequencing the genome of Marssonina brunnea reveals fungus-poplar co-evolution
Source: BMC Genomics. 2012 Aug 9;13:382. doi: 10.1186/1471-2164-13-382 (PMC3484023; doi:10.1186/1471-2164-13-382)
Supplement: Additional file 5 — Table S3. Top 20 protein families in M. brunnea that are the most significantly different from those of other fungal genomes including B. cinerea, S. sclerotiorum, M. grisea, and F. graminearum. [file 1471-2164-13-382-S5.doc]

Table S3 Top 20 protein families in *M. brunnea* that are the most significantly different from those of other fungal genomes including *B. cinerea, S. sclerotiorum, M. grisea, and F. graminearum.*

| Pfam ID | Family Name | *M. brunnea* | *B. cinerea* | *S. sclerotiorum* | *M. grisea* | *F.* | *p*-value |
| --- | --- | --- | --- | --- | --- | --- | --- |
|  |  |  |  |  |  | *graminearum* |  |
| PF01476 | LysM domain | 33 | 4 | 6 | 7 | 8 | 7.15E-05 |
| PF01408 | Oxidoreductase family, NAD-binding Rossmann fold | 8 | 2 | 2 | 1 | 2 | 0.00014 |
| TIGR02169 | SMC_prok_A: chromosome segregation protein SMC | 6 | 0 | 0 | 0 | 1 | 0.00018 |
| PF00076 | RNA recognition motif. (a.k.a. RRM, RBD, or RNP domain) | 54 | 38 | 34 | 35 | 37 | 0.000285 |
| TIGR02168 | SMC_prok_B: chromosome segregation protein SMC | 14 | 0 | 1 | 2 | 3 | 0.000301 |
| PF00125 | Core histone H2A/H2B/H3/H4 | 16 | 10 | 8 | 8 | 9 | 0.000625 |
| PF00534 | Glycosyl transferases group 1 | 8 | 5 | 4 | 4 | 4 | 0.000643 |
| PF03446 | NAD binding domain of 6-phosphogluconate dehydrogenase | 7 | 2 | 2 | 3 | 1 | 0.001172 |
| PF01753 | MYND finger | 17 | 8 | 4 | 7 | 7 | 0.001208 |
| PF00004 | ATPase family associated with various cellular activities (AAA) | 26 | 4 | 4 | 10 | 9 | 0.001237 |
| PF01757 | Acyltransferase family | 10 | 2 | 5 | 4 | 3 | 0.002085 |
| PF03169 | OPT oligopeptide transporter protein | 7 | 12 | 12 | 12 | 11 | 0.000318 |
| PF00005 | ABC transporter | 11 | 25 | 22 | 22 | 21 | 0.000923 |
| PF04909 | Amidohydrolase | 4 | 8 | 7 | 7 | 8 | 0.001208 |
| PF00722 | Glycosyl hydrolases family 16 | 10 | 20 | 18 | 17 | 20 | 0.001353 |
| PF00664 | ABC transporter transmembrane region | 12 | 18 | 17 | 18 | 20 | 0.00217 |
| PF00067 | Cytochrome P450 | 50 | 127 | 94 | 131 | 112 | 0.004289 |
| PF07992 | Pyridine nucleotide-disulphide oxidoreductase | 18 | 26 | 24 | 22 | 26 | 0.006533 |
| PF00698 | Acyl transferase domain | 2 | 6 | 5 | 6 | 4 | 0.006533 |
| PF01494 | FAD binding domain | 22 | 45 | 33 | 38 | 44 | 0.007621 |
