# Supplementary material for: Sequencing the genome of Marssonina brunnea reveals fungus-poplar co-evolution
Source: BMC Genomics. 2012 Aug 9;13:382. doi: 10.1186/1471-2164-13-382 (PMC3484023; doi:10.1186/1471-2164-13-382)
Supplement: Additional file 9 — Text S1. Additional Description. [file 1471-2164-13-382-S9.doc]

**Text S1: Additional Description**

### Analysis of repetitive sequences

RepeatModeler (v1.0.4) and RepeatMasker (v3.2.9) ([http://www.repeatmasker.org](http://www.repeatmasker.org/)) were used to search for low-complexity sequences, simple repeats with a unit size ranged from 1 to 6 bp, and known repeat elements in the genome sequences of *M. brunnea*, *B. cinerea* (strain B05.10), and *S. sclerotiorum* (strain 1980). RepeatModeler is a de novo tool that identifies repeat families, including RECON (<http://selab.janelia.org/recon.html>) and RepeatScout (http://bix.ucsd.edu/repeatscout/), which are de novo repeat finding programs.

The amounts of low-complexity sequences of the three fungi are given in additional file 4 Table S2, where the low-complexity sequence in *S. sclerotiorum* was found to be more than that in *M. brunnea* and *B. cinerea*. AT-rich repeats of *M. brunnea* were abundant as well as those of *B. cinerea* and *S. sclerotiorum*, accounting for approximately 25% of low-complexity regions. The amount of AT-rich repeats in *M. brunnea* was less than a half of that of *B. cinerea*, and less than one third of that of *S. sclerotiorum*. Besides AT-rich repeats, other types of low-complexity regions in *M. brunnea* were more than those in *B. cinerea* and *S. sclerotiorum*, such as A-rich, G-rich, GA-rich, and GC-rich (additional file 4 Table S2).

The amount of simple sequence repeats (SSRs) in the three fungi is given in additional file 17 Table S9. SSRs can serve as co-dominant molecular markers that are important to fungal genetics research for genotyping . The SSR content for *M. brunnea* was about 1.3% of the genome, more than that in *B. cinerea* and *S. sclerotiorum*. SSR sequences for each unit size in *M. brunnea* were also more common than in *B. cinerea* and *S. sclerotiorum*. Most SSR sequences in all three fungi have a unit size of 3–5 bp (75% in *M. brunnea*, 79% in *B. cinerea*, and 84% in *S. sclerotiorum*). Poly (AT)_n_ represents the most abundant class of SSR in these fungi, e.g., in *M. brunnea* with a total of 934 repeats accounting for 37 kb (Table 3). Interestingly, we found no short interspersed nuclear elements (SINE) in the genome of *M. brunnea*, *B. cinerea*, and *S. sclerotiorum*. *M. brunnea* contains more repeats than *B. cinerea* and *S. sclerotiorum*, especially LTRs (long terminal repeats). LTRs accounted for a total of 13,438 Kb in *M. brunnea*, ~80 times more than in *B. cinerea* and 37 times more than in *S. sclerotiorum*, accounting for 26% of the genome. LTR retrotransposons have been observed in fungi, plants, and animals , differ widely in their abundance and can promote genome rearrangements . LTR/Gypsy content accounted for ~17 % of the genome for *M. brunnea*, 0.4 % and 0.9 % of the genomes of *B. cinerea* and *S. sclerotiorum* (Table 3). There were 4,480 Kb of LTR/Copia found in the genome of *M. brunnea*.

For many species, the sequence of telomeric DNA consists of tandem arrays of 5'-TTAGGG-'3 repeats (http://telomerase.asu.edu/sequencestelomere.html). The repeat sequence unit in human telomeres is TTAGGG, which is the same as in several fungal species, such as *Neurospora crassa*, *Podospora anserina*, *M. grisea*, *Pestalotiopsis microspora*, and *Fusarium oxosporum* . We identified telomeric sequences by searching for the telomere repeat motif (TTAGGG)_n_ in *M. brunnea*, and found five potential sites. It is possible that some telomeric loci were not identified due to the failure to obtain the sequences of some regions from whole-genome sequencing.

### Protein Families

We identified protein families of other four fungi, *B. cinerea*, *S. sclerotiorum*, *M. grisea*, and *F. graminearum*, using the methods described above. Results from a *t*-test suggest that *M. brunnea* is remarkably different in the number of protein families from the other four fungal species (additional file 18 Table S10). Many protein families involved in transport were found in *M. brunnea*, such as MFS (major facilitator superfamily), ABC (ATP-binding cassette) and sugar transporters. MFS and ABC superfamilies are two of the largest protein families associated with fungal pathogenesis and a few MFS and ABC transporters may be involved in fungicide efflux . More AAA ATPases were found in *M. brunnea* than in *B. cinerea*, *S. sclerotiorum*, *M. grisea* and *F. graminearum* (additional file 18 Table S10). A few AAA ATPases are essential for normal morphology required for maintaining virulence, such as CaYLL34 .

We found 33 LysM (Pfam: PF01476) domain-containing proteins in the genome of *M. brunnea*, a number being much larger than for *B. cinerea* (4), *S. sclerotiorum* (6), *M. grisea* (7) and *F. graminearum* (8). LysM domain-containing secreted proteins are widely conserved in fungi, such as ECP6 from *Cladosporium fulvum* . Of these 33 LysM genes, 30 had no intron and 28 with only one exon were highly similar and predicted as secreted proteins and ranged in size from 142 to 151 amino acid (AA) residues. Given their abundance, the LysM proteins may play a key role in cellular processes of *M. brunnea*.

Cytochrome P450 (Pfam: PF00067) enzymes are a superfamily of heme-containing monooxygenases and play diverse roles in fungal biological processes, such as metabolizing the detoxification of plant defense response compounds . Fewer cytochrome P450 family proteins were found in the genome of *M. brunnea* (50) than *B. cinerea* (127), *S. sclerotiorum* (94), *M. grisea* (131), and *F. graminearum* (112). Many cytochrome P450 genes are highly conserved, such as *sirB*, *sirC*, and *sire* . Fifty putative cytochrome P450 proteins were clustered into 46 families by BLASTclust (ftp://ftp.ncbi.nih.gov/blast/documents/blastclust.html) using threshold limits of 40% identity and 80% length overlap .

*M. brunnea* also contains protein families that are different from those in *B. cinerea*, *S. sclerotiorum*, *M. grisea*, and *F. graminearum.* A total of 15 heterokaryon incompatibility (HET) proteins (Pfam: PF06985) were found in *M. brunnea*, much fewer than in *B. cinerea* (64), *S. sclerotiorum* (40), *M. grisea* (41), and *F. graminearum* (94). This family of proteins, which seems to belong exclusively to ascomycete fungi, consists of a conserved region of approximately 150 residues . The Het-s protein of *P. anserina* is prion proteins involved in heterokaryon incompatibility . The fusion cell formed by two strains that differ allelically at the het loci is compartmentalized and undergoes a form of programmed cell death (PCD) termed vegetative or heterokaryon incompatibility (HI) . Heterokaryon incompatibility may play a key role in limiting heterokaryosis for genetic recombination . When fungi encounter antifungal agents, such as development signal and stress factors, they can undergo PCD . NACHT family (Pfam: PF05729) proteins, which are nucleotide triphosphatases (NTPases), may be involved in the regulation of the immune response and related apoptosis or to PCD in animals and fungi. There were a few NACHT proteins found in the genome of *B. cinerea* (7), *S. sclerotiorum* (7), *M. grisea* (11), and *F. graminearum* (29), but curiously none in *M. brunnea*.

### Genes involved in pathogenesis

Fungal infection of plants is a very complex process, including recognition, invasion, competition, pathogenesis and so on. Many genes are involved in this process, most of which are homologs in *M. brunnea*. Unlike well-studied *M. grisea* and *B. cinerea*, there is no information about the pathogenic mechanisms by *M. brunnea*. This will change with the completion of the draft sequence of the *M. brunnea* genome which allows pathogenic genes to be identified at the genome level. Phi-base (pathogen-host interaction database) is a database that collects pathogenicity, virulence, and effector genes from fungi, oomycetes, and bacterial pathogens . A total of 793 predicted genes shared homology to 622 of 924 genes in Phi-base (v3.2), when we used BLASTP with an E-value of <1E-10. Additional file 6 Table S3 shows the number of proteins with more than 10 homologs from *M. brunnea*. Genes in phi-base are not divided in detail by function, so we attempted to classify putative pathogenicity genes in *M. brunnea* for understanding the interaction of plant and fungus.

#### Genes involved in recognizing the host and signal pathways

The recognition of a host by a pathogen triggers numerous signaling cascades, leading to the expression of genes involved in entry into host cells through the formation of specialized infection structures . The first step of invasion is the recognition of the host surface. PTH11, a novel transmembrane protein of *M. grisea*, is required for host surface recognition through a cell-cortex G-protein-coupled receptor (GPCR) and mediates cAMP signaling or functions in an overlapping signaling pathway . Besides, *PTH11* is an upstream effector of appressorium differentiation in response to surface cues . In *M. brunnea*, we found five genes (additional file 6 Table S3) homologous to PTH11 (Best e-value: 5E-69), but it is unknown which of these genes functions similarly as PTH11 does.

Signal transduction cascades regulating fungal development and virulence are remarkably conserved in distantly related fungi, especially those involving cAMP-dependent protein kinase and mitogen-activated protein kinase (MAPK) . Involved in the cAMP-dependent pathway , cyclic AMP signaling is required for the induction of appressorium formation and the turgor-driven process that leads to plant infection. *BCG1*, one of the three Gα subunit-encoding genes in *B. cinerea*. The genome of *M. brunnea* revealed a gene that encodes a BCG1-like protein, a relatively conserved protein found in most fungi. The importance of this common pathway in vegetative growth and pathogenicity was confirmed by the inactivation of the adenylate cyclase gene, *BAC*, from *Botrytis cinerea* . In addition, *BCG1* controls other genes, including botryoidally protease *BcBOT1* and CWDE (cell wall degrading enzyme) genes . The botrydial gene (including *BOT1*, which was also identified in *M. brunnea*) is controlled by the calcineurin-dependent pathway . Calcineurin phosphatase plays an essential role in fungal morphogenesis and virulence and was shown to be involved in *M. grisea* and *B. cinerea* appressorium formation .

Signaling networks of filamentous fungi usually contain three MAPK cascades . The corresponding MAPKs, including *MPK1*, *HOG1*, *PBS2*, *SSK2*, *SSK22*, and *STE11* have been identified in the *M. brunnea* genome sequence. *HOG1* is the ortholog of the *B. cinerea* *SAK1*, and *MPK1* is the ortholog of the yeast *FUS3/KSS1* and of the *M. grisea* virulence factor PMK1. The PMK1 pathway may be well conserved in fungal pathogens for regulation of plant infection processes through host surface sensing and appressorium formation . Another well-studied MAPK module is the high osmolarity glycerol (HOG) pathway in the model yeast *S. cerevisiae*, which responds to changes in external osmolarity and oxidative stresses. There are three levels of MAPK cascades. *SSK2*, *SSK22*, and *STE11* are the MAPKKK genes, activation of which phosphorylates *PBS2*, a MAPKK protein. The signal can be transferred to the downstream *HOG1* MAPK by phosphorylation of conserved threonine and tyrosine residues . However, in contrast to the HOG1 pathway in *S. cerevisiae*, *HOG1* in *C*. *albicans* is regulated by a single MAPKKK, *SSK2* . Therefore, the HOG1 MAPK signaling network has diverged significantly in different fungi. These MAPKKK genes were all identified in *M. brunnea*, but how they form an HOG1-like MAPK network requires further investigation.

#### Genes affecting fungal cell wall biosynthesis and infection structure

Fungal structures, including the cell wall, appressorium, and hyphae, are important for development and pathogenicity. A large number of genes are involved in the biosynthesis of these structures. Chitin synthases catalyze the synthesis of chitin, which is the major component of fungal cell walls, and have been studied in many fungi. Disruption of the chitin synthase class 1 gene (*CHS1*) and class 3 gene (*CHS3a*) are involved in the biosynthesis of fungal cell walls, and disruption of *CHS1* or *CHS3a* will significantly affects the synthesis of chitin and drastically reduces *B. cinerea* virulence . Both of these chitin synthases have homologous genes in *M. brunnea* (additional file 11 Table S4).

Fungi differentiate appressoria for successful penetration. After adhering to the host surface, appressoria will create a high turgor pressure that allows the penetration peg to penetrate the host epidermis . Appressorium formation is induced by some specific substance secreted by the host. As appressoria was found during the growing and infection process of many fungi . We also observed appressorium formation in *M. brunnea* while it was growing (Fig. 1). Appressorium formation involves a large number of genes, and disruptions in many of them will arrest fungal growth and pathogenesis. In *M. grisea*, *PTH11* was identified as an upstream activator of cAMP-mediated signaling and a crucial pathogenicity gene for efficient appressorium formation, as discussed above. Disruption of *PTH11* in *M. grisea* resulted in non-pathogenic mutants that were unable to recognize the host plant and produce normal appressoria . We also identified a putative gene homologous to *ORP1* from *M. grisea.* The disruption of *ORP1* caused the reduction of pathogenicity after appressorium formation .

The penetration peg, also called the penetration hypha, forms at the base of appressoria. This narrow cylindrical structure breaks the host plant cuticle and cell wall . The gene *PLS1*, encodes a putative integral membrane protein in *M. grisea*. Morphological, cytological, and structural analyses showed that *PLS1* is required for the differentiation of the appressorium penetration peg . We have identified one homologous genes of *PLS1* in *M. brunnea* (additional file 6 Table S3). In *M. grisea*, another gene, *PDE1*, encodes a protein that has homology to aminophospholipid translocase group of P-type ATPase (AAA ATPase). The expression pattern and phenotype of *PDE1* mutants suggest that *PDE1* is essential for development of penetration hyphae and proliferation of the fungus . We identified five genes in *M. brunnea* that had significant homology with *PDE1*.

Nicotinamide adenine dinucleotide (NADPH) oxidases are involved in the differentiation of fungi. Two NADPH oxidases genes, *NOXA* and *NOXB*, have been studied in *B. cinerea*. Single and double disruption mutants of these two genes in *B. cinerea* indicated that *NOXA* and *NOXB* are required for the formation of sclerotia and have a great impact on pathogenicity . We identified two NADPH oxidases genes in M. *brunnea*, *MbNOXA* and *MbNOXB*, which show significant homology to *NOXA* and *NOXB*.

#### Genes involved in degradation of the plant cuticle and cell wall

The plant cell wall is a major barrier to infection by fungal pathogens; therefore, CWDEs are required for penetration and spread of potential pathogens. Successful penetration should be supported by enzymes capable of degradation of plant cuticle and cell wall. Therefore, phytopathogenic fungi secrete a large number of degradative enzymes. Microscopic observation of the process of *M. brunnea* penetrating poplar leaves revealed that the hyphae of *M. brunnea* can penetrate poplar leaves cuticle and cell wall without obvious distortion, suggesting that little physical force is necessary and that several hydrolytic enzymes are involved in penetration .

We identified 599 secreted proteins, several of which were predicted to encode hydrolytic enzymes for the degradation of plant cuticle and cell wall (additional file 6 Table S3). For example, six genes had a significant homology with the cutinase gene *CUTA*, which is expressed by *B. cinerea* during the penetration of tomato . One of these six genes (M6_03198) was significantly up-regulated at 96 hpi (hour post inoculation) (additional file 11 Table S4), indicating that it may play a key role in breaching the cutin layer of the host leaf. Five genes in *M. brunnea* putatively encode pectinlyase, which can degrade pectin, the major component of host cell wall. The homologous gene, *PL1*, is required for full virulence of *F. oxysporum* (additional file 6 Table S3).

Cellulose is the main component of plant biomass. Cellulases in fungi can facilitate complete cleavage so that fungi can successfully infect and obtain nutrients from the plant host. Cellulases can be divided into three types: type I, endo-β-1,4-glucanase (EC 3.2.1.4); type II, exo-β-1,4-glucanase (cellobiohydrolase, CBH; EC 3.2.1.91); and type III, β-glucosidase (EC 3.2.1.21) . Sequence alignment and phylogenetic analysis showed that two CBHs, two endo-β-1,4-glucanases, and four β-glucosidases are present in *M. brunnea* (additional file 11 Table S4)*,* suggesting that all three types of cellulase are required for virulence of *M. brunnea.* One of the two CBHs, M6_06371, may operate early in the infection process, as reported by a study of *M. brunnea* secreted proteins . Homologous genes encoding many other enzymes, such as endoglucanases and exopolygalacturonases, which are required for degrading the plant cuticle and cell wall, were also found in the *M. brunnea* genome (additional file 6 Table S3).

#### Genes for pathogen protection mechanisms during infection process

Host defense responses are initiated when a pathogen is recognized by its host. Coping with these responses is the main challenge that pathogens confront. Fungi have mechanisms to escape detection, to avoid induction of the host immunity systems, and to deal with the defense responses when they occur (Fig. 4). Pathogen-associated molecular pattern genes (PAMPs) like chitin can trigger plant immunity. *C. fulvum* *ECP6* encodes a small secreted protein that sequesters chitin oligosaccharides to prevent the elicitation of host defense responses . Two highly similar homologs were identified in *M. brunnea*.

Hosts also can secret toxins when they are invaded. These toxins are serious threats to the growth and development of the pathogen. The *ABC1*-encoded protein of *M. grisea*  allows efflux of toxic antimicrobial compounds and this lets the fungus build up resistance to the host . The *ABC3* gene (Fig. S3) from *M. grisea* is required to withstand oxidative stress and the host-specific adverse environment . In *M. brunnea*, six such genes were found. *ATRB*, an ABC transporter in *B. cinerea* induced by camalexin, also acts as an efflux pump of toxic compounds . Disruption of these genes reduces pathogenicity. Seven *ATRB* homologs were identified in *M. brunnea* (additional file 6 Table S3).

Pathogens also produce enzymes to degrade antifungal compounds. AVENACINASE, a secreted saponin-degrading enzyme of *G. graminis,* can detoxify the triterpenoid oat root saponin, avenacin A-1. AVENACINASE has been proven to be essential for pathogenicity . In *M. brunnea,* we found three homologs of the *AVENACINASE* gene. Tomatinase is another Saponinase found in *F. oxysporum*, encoded by *TOM1*, of which there is one homolog in *M. brunnea* .

Pathogens can product some substances that [contribute](http://www.nciku.cn/search/en/contribute) to infection. As these substances may be harmful to both the pathogen and its host, pathogens have developed [mechanism](http://www.nciku.cn/search/en/mechanism)s to avoid injury. Metabolic alteration of the toxic compound is one way to protect fungi from their own toxins. For example, *Fusarium graminearum* and *Fusarium culmorum* can produce the trichothecene mycotoxin, deoxynivalenol (DON) to inhibit protein synthesis in the host. To protect itself from DON, this fungus has a gene called *TRI101*, which encodes trichothecene 3-O acetyltransferase . Two homologs of *TR1101* were found in *M. brunnea*.

#### Roles of genes in fungal toxin biosynthesis

Toxins are produced by fungi to kill host cells or disable host cellular functions. They can be non-host-specific, which are effective in unrelated plants, or host specific, which affect only a certain species (Fig. 5). Fungi have many genes that control the biosynthesis, regulation, and export of toxins. Cercosporin is a light-activated non-host-specific toxin. It can produce activated oxygen species that cause damage to the host plasma membrane through peroxidation . The polyketide synthase gene (*CTB1*) of *Cercospora nicotianae* plays a key role in cercosporin biosynthesis. Mutants in *CTB1* show reduced virulence . Three homologs of *CTB1* were found in *M. brunnea* (additional file 6 Table S3). There were also some other genes related to Cercosporin biosynthesis, such as CFP, which encodes a cercosporin transporter in *Cercospora kikuchii* , has five homologs in *M. brunnea.* *CZK3*, which has one homolog in *M. brunnea*, act as a MAP kinase kinase kinase in *Cercospora zeae-maydis* to regulate cercosporin biosynthesis. Disruption of CZK3 reduces the production of cercosporin. Among them, *CTB1*and *CZK3* in *M. brunnea* are highly similar to their counterparts in *Cercospora nicotianae.*

Genes encoding host-specific toxins, such as victorin, HC-toxin, AK-toxin, AM-toxin, and ACT-toxin, are often clustered. For example, HC-toxin inhibits the host histone deacetylase, thereby distorting the proper regulation of defense gene activation. *HTS1* encodes a multifunctional cyclic peptide synthetase, which is involved in the biosynthesis of HC-toxin. Disruption of *HTS1* in *Cochliobolus carbonum* resulted in inability to produce HC-toxin and loss of pathogenicity . Besides *HTS1*, *TOXC* and *TOXF* are also essential for toxin biosynthesis and pathogenicity. All of them have very similar counterparts in *M. brunnea*.

*Alternaria alternata* attacks the host Japanese pear through secreting a host-specific toxin called the AK-toxin. *AKT1*, encoding a series of carboxyl-activating enzymes, and *AKT2* are involved in the biosynthesis of the AK-toxin . Homologs of *AKT1* and *AKT2* were found in *M. brunnea*. As in *A. alternata*, the homologous genes found in *M. brunnea* were also clustered.

AM-toxin is a cyclic peptide host-specific toxin used in the infection process of the *A. alternata* apple pathotype. The *AMT* gene is required for the biosynthesis of the AM-toxin . A homolog of *AMT* exists in *M. brunnea*. *ACTTS* and *ACTTS3* are essential genes for ACT-toxin biosynthesis. Mutants of them lack ACT-toxin production and pathogenicity. Two genes with a high similarity to them were found separately in *M. brunnea*.

#### Roles of fungal genes in nutrient acquisition

Fungi need to adapt their nutrient status to the new environment during the invasion and growth phase within the host . Most of the known genes with nutritional acquisition functions in other fungi are present in *M. brunnea*. These include genes involved in acquisition and regulation of sugars, minerals, amino acids, and fatty acids.

Sugars are crucial nutritional substances for successful pathogenesis. The *U. maydis SRT1* gene encodes a high-affinity sucrose transporter, which is characterized as a virulence factor. It allows the utilization of sucrose without extracellular hydrolysis, preventing elicitation of the plant defense response . There are five homologs of *SRT1* in *M. brunnea* (additional file 6 Table S3)*.* Minerals such as iron are also indispensable to fungi. There are two strategies for pathogens to obtain iron, the siderophore-mediated iron uptake system and the reductive iron assimilation system. Small iron-chelating peptides called Siderophores are helpful to directly acquire iron. *SIB1* from *S. pombe* and *SIDC* from *Aspergillus nidulans* have been functionally characterized, and are involved in siderophore biosynthesis . Knockout of these genes can cause inability of the fungus to acquire iron. In *M. brunnea*, two homologs of *SIB1* and one homolog of *SIBC* are present. A GATA transcription factor, *URBS1*, negatively controls the expression of the *SID1* gene in *U. maydis* in the presence of iron, indicating the existence of a regulatory [mechanism](http://www.nciku.cn/search/en/mechanism) . A homolog of *URBS1* in *M. brunnea* was identified.

Oxidases and permeases form a complex and are a part of the other high-affinity iron uptake system. *FER1* encodes iron multicopper oxidases and *FER2* encodes a high-affinity iron permease in *U. maydis,* and they have three homologs and one homolog in *M. brunnea*, respectively. Mutations to them in *U. maydis* reduce virulence . *PIG2*, encoding a putative amino acid permease in *U. fabae*, is haustorium-specifically expressed . Five homologs of *PIG2* were found in *M .brunnea*.

Peroxisomes are single membrane-bound organelles involved in metabolic pathways in eukaryotes, such as the glyoxylate pathway related to fatty acids metabolism . *A. alternata* peroxin gene *PEX6* encodes a peroxin essential for peroxisome biogenesis. Mutants of *PEX6* can cause nutritional deficiency and loss of pathogenicity . *ClaPEX6*, an ortholog of *PEX6* from *C. lagenarium*, plays a critical role in peroxisomal metabolism and appressorium- mediated infection , whose mutants form a small and nonmelanized appressoria and weaken the ability to generate penetration hyphae . In *M. brunnea*, we have found four *PEX6* homologs.

### Secreted proteins and effectors

Plant pathogenic fungi require secreted proteins to alter their environment and host for facilitation of infection . Effectors which play a key role in disease symptom development are small proteins secreted by plant pathogenic fungi . There are a great number of secreted proteins involved in fungus-plant interactions, accounting for ~15% of the total proteome in fungal/oomycete genomes . The majority of secreted proteins commonly have an N-terminal signal peptide that is removed by specialized signal peptidases during the transport process of the secreted protein . However, many membrane proteins also possess a signal peptide at their N-termini . Of 10,027 gene models in *M. brunnea*, 872 *M. brunnea* genes contain a potential signal peptide by analysis with SignalP (v3.0), 599 (6%) of the predicted genes encoded proteins with a putative signal peptide but without a transmembrane region (TM) as determined by TMHMM (v2.0). Secreted proteins accounted for 6.6%, 13.3%, 4.6%, and 4.3% of the proteomes in *U. maydis*, *M. grisea*, *B. cinerea*, and *S. sclerotiorum*, respectively (additional file 12 Table S5, additional file 19 Table S11). Many secreted proteins are conserved in fungi. Three hundred fourty four of the predicted secreted proteins in *M. brunnea* were aligned to secretory proteins of six fungi (*U. maydis*, *M. grisea*, *B. cinerea*, *S. sclerotiorum*, *S. cerevisiae*, and *S. pombe*) from FunSecKB (Fungal Secretome Knowledge base, <http://proteomics.ysu.edu/secretomes/fungi.php>), by BLASTP with a cutoff E-value <1e-5.

The lengths of 599 predicted secreted proteins in *M. brunnea* range from 55 amino acid（AA） to 1033 AA，averaging 303 AA. These numbers are similar in *B. cinerea* ranging from 30 to 1280 AA and averaging 362 AA and *S. sclerotiorum* ranging from 33 to 1761 AA and averaging 384 AA (Fig. S5). Of 15 secreted proteins, seven with LysM domain have been identified in our previous studies by two-dimensional gel electrophoresis (2-DE) and tandem mass spectrometry (MS/MS) . The 15 secreted proteins were also found in the 599 predicted secreted proteins. In addition, 12 predicted proteins were aligned to 14 peptide fragments obtained by 2-DE and MS/MS spectrum, of which 11 were identified as secreted proteins.

Cutinases in fungal pathogens can hydrolyze cutin, the structural component of cuticles, which is the first barrier of a plant to resist pathogenic fungi . Eight putative secreted proteins with a cutinase (PF01083) domain were identified in *M. brunnea* (additional file 12 Table S11). Pectate lyase (EC 4.2.2.2), a secreted enzyme, is required for the maceration and soft-rotting of plant tissue by cleaving cell wall pectic polymers . *PELB*, a pectate lyase from *Colletotrichum gloeosporioide*, plays an important role in the colonization of avocado fruit . Eight putative secreted proteins with the Pec_lyase_C (PF00544) motif and five putative secreted proteins with pectate lyase (PF03211) motifs were identified in *M. brunnea,* respectively (additional file 19 Table S11)*.*There are a great number of pectate lyases-like sequences in plants: *Arabidopsis* encodes 27 such proteins . In addition, eight putative secreted proteins containing GDSL-like lipase/acylhydrolase (PF00657) were found in *M. brunnea* (additional file 19 Table S11)*.* In several reports, lipolytic enzymes and lipases have been considered as virulence factors in plant pathogenic fungi .

Twenty nine putative secretory proteins belong to LysM family (additional file 19 Table S11), of which twenty eight proteins were highly similar (Fig. 6). A few LysM domain-containing secreted proteins from plant pathogenic fungi are important. For example, Ecp6 may be responsible for chitin binding . Interestingly, many LysM RLK family genes from plants are responsible for chitin signaling in plant innate immunity, such as LysM RLK1 from *A. thaliana* . LysM RLK1 can directly bind to chitin in the fungal cell wall . In addition, some LysM-containing genes have been found in poplar . Chitin is the major component of cell wall in fungi, but not in plants. LysM receptor kinases from plants bind to or interact with chitin in the fungal cell wall, resulting in plant basal immunity . If pathogenic fungi suppress or resist plant basal immunity, they need to avoid recognition by plants . We hypothesize that, as in *C. fulvum*, *M. brunnea* might suppress plant basal immunity by secreting fungal LysM proteins that can compete with host LysM receptor kinases for the binding of fungal chitin .

Effectors are secreted by microbial to accelerate disease development or suppress the basal immunity in the plants, they are often small and cysteine-rich secreted proteins . Of 257 putative proteins identified with more than six cysteines, 42 shared homology with virulence proteins in Phi-base. For instance, *HopI1*, a virulence effector from *Pseudomonas syringae*, plays a crucial role in enhancement of pathogenicity or inhabitation of plant defenses . Interestingly, 28 secreted and highly similar LysM proteins contained more than six cysteines. More than half of 77 putative secreted and virulence proteins were putative cysteine-rich effectors, these possibly playing important roles in promoting its virulence or repressing host basal defenses.

### Mating and meiosis

Like many other fungi, *M. brunnea* is mostly haploid. Each hyphal segment contains one haploid nucleus with three chromosomes (Fig. 1). *M. brunnea* reproduces asexually by generating spores with single haploid nuclei by mitosis. Conidiophores partition asexual spores in longitudinal chains. When mature, conidia are released in large numbers and germinate to produce new mycelia. So far, the sexual stage of *M. brunnea f. sp. multigermtubi* obtained from the eastern China (including Shandong, Jiangsu, Henan, Shanxi, Jilin Provinces, and Beijing) have not been observed, the same as in New Zealand Farm Forestry (<http://www.nzffa.org.nz/farm-forestry-model/the-essentials/forest-health-pests-and-diseases/diseases/Marssonina/Poplar-anthracnose>). Furthermore, in our previous research, a sexual cycle was not observed in this species isolate from South China.

To confirm whether *M. brunnea* has a mating cycle and performs meiosis, we compiled a list of mating type loci (MAT) and related genes in *S. sclerotiorum and B. cinerea,* which belong to Ascomycota as *M. brunnea*. The majority of genes were conserved and easily identifiable using BLAST searches (additional file 11 Table S4). Usually, MAT contain two key domains, alpha- and high mobility group (HMG)-domain, encoding MAT genes. These loci are critical determinants of reproductive mode. The majority of homothallic species contain both alpha and HMG domain-encoding genes in one chromosome, like *S. sclerotiorum* , or in separate chromosomes, like *Neosartorya fischeri* . The heterothallic species contain either a alpha or a HMG idiomorph, like *B. cinerea* . All of these MAT are missing in *M. brunnea*. Furthermore, the alpha-factor modification (*STE16*) and other genes including *ACT1, BUD6, FAR1, AXL1,BIM1* were not present . This phenomenon is similar with *L. elongisporus*, who may not have a sexual cycle or mating independently of *MAT/MTL*.

To identified whether *M. brunnea* perform sex cycle and meiosis, some of genes related to synapsis and meiotic pathway have been compared with 7 kind of yeasts, *C. albicans, Lodderomyces elongisporus, C. guilliermondii, D. hansenii, S. sclerotiorum, S. cerevisiae*, and *S. pombe,* who play a mode role in fungi (additional file 11 Table S4). In this section, *M. brunnea* also exhibits some specific gene losses concerned with synapsis in the early phase of meiosis. They lack many genes about the synaptonemal complex (SC), and the synapsis initiation complex (SIC) components, including *HOP1, RED1* , *MAM1, MND2 , ZIP1*, *ZIP3* and *ZIP2* *.* In that *HOP1*and *RED1* plays a major role in the formation of the SC *.* Although the complete absence of the SC is rare in eukaryotes, it has been reported that in *S. pombe*  has evolved another pathway called Linear elements (LinEs) to reduce SCs. *S. pombe* contains only three chromosome pairs and therefore may have little need for SCs during homolog pairing . *REC10* and *REC8* involved in LinEs. *REC10* can form central LinEs and *REC8* is a meiosis-specific component of the cohesin complex required for LinE formation . But in *M. brunnea*, neither SC associated components (including *RED1*, *HOP1* and *MEK1*), nor the major components of LinEs (including *REC10* and *REC8*) were missing at all. Therefore, we can hypothesize that the SC and LinEs are absent in *M. brunnea.*

In contrast to the mating and synapsis related genes, two core genes required for meiosis in two different pathway, *DMC1*and *RAD51*, were found in *M. brunnea* . *DMC1* appears to be involved in homology searching and strand exchange during meiotic recombination, and may contribute to partner choice . However, recent experiments have indicated that *DMC1* does not itself supply specificity to the strand invasion reaction. Two additional genes, *MEI5* and *SAE3*, which were not identified in *M. brunnea*, are necessary for efficient formation of DMC1-containing nucleoprotein filaments *in vivo* . So the DMC1 dependent pathway seems not exist in *M. brunnea*, and meiosis may involve the *RAD51* pathway. [Whereas](javascript:;), *RAD51* is unnecessary for meiotic recombination, it also works in double-strand break repair (DSBR) in vegetative or somatic cells . Besides, *RAD51* and *DMC1* exhibit extensive colocalization dependent on initiation of recombination by *SPO11* , whereas the homologue of *SPO11* does not exist in *M. brunnea*. Nevertheless, some genes correlated with meiosis are present in *M. brunnea*. For example, *RIM11* and *IME2*, which are required for the transcription of middle meiosis-specific genes , and *CDC5* and *CDC14*, which are cell cycle regulators .

This is the first report to investigate whether *M. brunnea* has a mating and sexual cycle. Two thirds of the genes related to sexual reproduction and meiosis were not present in *M. brunnea*; only 40 such homologues were found. In general, the sex-related genes that do not exist in *M. brunnea* function in mating, synaptonemal complex formation, and meiotic processes. The functions of the genes that do exist involve regulation as transcription factors or supplementaries in syngenesis. Since mating and meiosis were not observed, we can hypothesize that *M. brunnea* does not have a sexual cycle.

References

1. Steimel J, Chen W, Harrington TC (2005) Development and characterization of microsatellite markers for the poplar rust fungi Melampsora medusae and Melampsora larici-populina. Mol Ecol Notes 5: 484-486.

2. Miller K, Lynch C, Martin J, Herniou E, Tristem M (1999) Identification of multiple Gypsy LTR-retrotransposon lineages in vertebrate genomes. J Mol Evol 49: 358-366.

3. Hua-Van A, Le Rouzic A, Maisonhaute C, Capy P (2005) Abundance, distribution and dynamics of retrotransposable elements and transposons: similarities and differences. Cytogenet Genome Res 110: 426-440.

4. Bennetzen JL (2005) Transposable elements, gene creation and genome rearrangement in flowering plants. Curr Opin Genet Dev 15: 621-627.

5. Schechtman MG (1990) Characterization of telomere DNA from Neurospora crassa. Gene 88: 159-165.

6. Javerzat JP, Bhattacherjee V, Barreau C (1993) Isolation of telomeric DNA from the filamentous fungus Podospora anserina and construction of a self-replicating linear plasmid showing high transformation frequency. Nucleic Acids Res 21: 497-504.

7. Teixeira MT, Gilson E (2005) Telomere maintenance, function and evolution: the yeast paradigm. Chromosome Res 13: 535-548.

8. Long DM, Smidansky ED, Archer AJ, Strobel GA (1998) In vivo addition of telomeric repeats to foreign DNA generates extrachromosomal DNAs in the taxol-producing fungus Pestalotiopsis microspora. Fungal Genet Biol 24: 335-344.

9. Powell WA, Kistler HC (1990) In vivo rearrangement of foreign DNA by Fusarium oxysporum produces linear self-replicating plasmids. J Bacteriol 172: 3163-3171.

10. Coleman JJ, Mylonakis E (2009) Efflux in fungi: la piece de resistance. PLoS Pathog 5: e1000486.

11. Reimann S, Deising HB (2005) Inhibition of efflux transporter-mediated fungicide resistance in Pyrenophora tritici-repentis by a derivative of 4'-hydroxyflavone and enhancement of fungicide activity. Appl Environ Microbiol 71: 3269-3275.

12. Melo AS, Padovan AC, Serafim RC, Puzer L, Carmona AK, et al. (2006) The Candida albicans AAA ATPase homologue of Saccharomyces cerevisiae Rix7p (YLL034c) is essential for proper morphology, biofilm formation and activity of secreted aspartyl proteinases. Genet Mol Res 5: 664-687.

13. de Jonge R, van Esse HP, Kombrink A, Shinya T, Desaki Y, et al. (2010) Conserved fungal LysM effector Ecp6 prevents chitin-triggered immunity in plants. Science 329: 953-955.

14. Degtyarenko KN, Archakov AI (1993) Molecular evolution of P450 superfamily and P450-containing monooxygenase systems. FEBS Lett 332: 1-8.

15. Wittstock U, Gershenzon J (2002) Constitutive plant toxins and their role in defense against herbivores and pathogens. Curr Opin Plant Biol 5: 300-307.

16. Gardiner DM, Cozijnsen AJ, Wilson LM, Pedras MS, Howlett BJ (2004) The sirodesmin biosynthetic gene cluster of the plant pathogenic fungus Leptosphaeria maculans. Mol Microbiol 53: 1307-1318.

17. Cuomo CA, Guldener U, Xu JR, Trail F, Turgeon BG, et al. (2007) The Fusarium graminearum genome reveals a link between localized polymorphism and pathogen specialization. Science 317: 1400-1402.

18. Hutchison E, Brown S, Tian C, Glass NL (2009) Transcriptional profiling and functional analysis of heterokaryon incompatibility in Neurospora crassa reveals that reactive oxygen species, but not metacaspases, are associated with programmed cell death. Microbiology 155: 3957-3970.

19. Balguerie A, Dos Reis S, Ritter C, Chaignepain S, Coulary-Salin B, et al. (2003) Domain organization and structure-function relationship of the HET-s prion protein of Podospora anserina. EMBO J 22: 2071-2081.

20. Hall C, Welch J, Kowbel DJ, Glass NL (2010) Evolution and diversity of a fungal self/nonself recognition locus. PLoS One 5: e14055.

21. Espagne E, Balhadere P, Penin ML, Barreau C, Turcq B (2002) HET-E and HET-D belong to a new subfamily of WD40 proteins involved in vegetative incompatibility specificity in the fungus Podospora anserina. Genetics 161: 71-81.

22. Fedorova ND, Badger JH, Robson GD, Wortman JR, Nierman WC (2005) Comparative analysis of programmed cell death pathways in filamentous fungi. BMC Genomics 6: 177.

23. Winnenburg R, Urban M, Beacham A, Baldwin TK, Holland S, et al. (2008) PHI-base update: additions to the pathogen host interaction database. Nucleic Acids Res 36: D572-576.

24. Dean RA (1997) Signal pathways and appressorium morphogenesis. Annu Rev Phytopathol 35: 211-234.

25. Kulkarni RD, Thon MR, Pan H, Dean RA (2005) Novel G-protein-coupled receptor-like proteins in the plant pathogenic fungus Magnaporthe grisea. Genome Biol 6: R24.

26. DeZwaan TM, Carroll AM, Valent B, Sweigard JA (1999) Magnaporthe grisea pth11p is a novel plasma membrane protein that mediates appressorium differentiation in response to inductive substrate cues. Plant Cell 11: 2013-2030.

27. Lengeler KB, Davidson RC, D'Souza C, Harashima T, Shen WC, et al. (2000) Signal transduction cascades regulating fungal development and virulence. Microbiol Mol Biol Rev 64: 746-785.

28. Xu JR (2000) Map kinases in fungal pathogens. Fungal Genet Biol 31: 137-152.

29. Schumacher J, Viaud M, Simon A, Tudzynski B (2008) The Galpha subunit BCG1, the phospholipase C (BcPLC1) and the calcineurin phosphatase co-ordinately regulate gene expression in the grey mould fungus Botrytis cinerea. Mol Microbiol 67: 1027-1050.

30. Klimpel A, Gronover CS, Williamson B, Stewart JA, Tudzynski B (2002) The adenylate cyclase (BAC) in Botrytis cinerea is required for full pathogenicity. Mol Plant Pathol 3: 439-450.

31. Schulze Gronover C, Schorn C, Tudzynski B (2004) Identification of Botrytis cinerea genes up-regulated during infection and controlled by the Galpha subunit BCG1 using suppression subtractive hybridization (SSH). Mol Plant Microbe Interact 17: 537-546.

32. Viaud M, Brunet-Simon A, Brygoo Y, Pradier JM, Levis C (2003) Cyclophilin A and calcineurin functions investigated by gene inactivation, cyclosporin A inhibition and cDNA arrays approaches in the phytopathogenic fungus Botrytis cinerea. Mol Microbiol 50: 1451-1465.

33. Fox DS, Heitman J (2002) Good fungi gone bad: the corruption of calcineurin. Bioessays 24: 894-903.

34. Viaud MC, Balhadere PV, Talbot NJ (2002) A Magnaporthe grisea cyclophilin acts as a virulence determinant during plant infection. Plant Cell 14: 917-930.

35. Tatebayashi K, Takekawa M, Saito H (2003) A docking site determining specificity of Pbs2 MAPKK for Ssk2/Ssk22 MAPKKKs in the yeast HOG pathway. EMBO J 22: 3624-3634.

36. O'Rourke SM, Herskowitz I (2004) Unique and redundant roles for HOG MAPK pathway components as revealed by whole-genome expression analysis. Mol Biol Cell 15: 532-542.

37. Cheetham J, Smith DA, da Silva Dantas A, Doris KS, Patterson MJ, et al. (2007) A single MAPKKK regulates the Hog1 MAPK pathway in the pathogenic fungus Candida albicans. Mol Biol Cell 18: 4603-4614.

38. Soulie MC, Perino C, Piffeteau A, Choquer M, Malfatti P, et al. (2006) Botrytis cinerea virulence is drastically reduced after disruption of chitin synthase class III gene (Bcchs3a). Cell Microbiol 8: 1310-1321.

39. Choquer M, Fournier E, Kunz C, Levis C, Pradier JM, et al. (2007) Botrytis cinerea virulence factors: new insights into a necrotrophic and polyphageous pathogen. FEMS Microbiol Lett 277: 1-10.

40. Bechinger C, Giebel KF, Schnell M, Leiderer P, Deising HB, et al. (1999) Optical measurements of invasive forces exerted by appressoria of a plant pathogenic fungus. Science 285: 1896-1899.

41. Deising HB, Werner S, Wernitz M (2000) The role of fungal appressoria in plant infection. Microbes Infect 2: 1631-1641.

42. Villalba F, Lebrun MH, Hua-Van A, Daboussi MJ, Grosjean-Cournoyer MC (2001) Transposon impala, a novel tool for gene tagging in the rice blast fungus Magnaporthe grisea. Mol Plant Microbe Interact 14: 308-315.

43. Bourett TM, Howard RJ (1990) In vitro development of penetration structures in the rice blast fungus Magnaporthe grisea. Canadian Journal of Botany 68: 329-342.

44. Clergeot PH, Gourgues M, Cots J, Laurans F, Latorse MP, et al. (2001) PLS1, a gene encoding a tetraspanin-like protein, is required for penetration of rice leaf by the fungal pathogen Magnaporthe grisea. Proc Natl Acad Sci U S A 98: 6963-6968.

45. Balhadere PV, Talbot NJ (2001) PDE1 encodes a P-type ATPase involved in appressorium-mediated plant infection by the rice blast fungus Magnaporthe grisea. Plant Cell 13: 1987-2004.

46. Balhadère PV, Foster AJ, Talbot NJ (1999) Identification of Pathogenicity Mutants of the Rice Blast Fungus Magnaporthe grisea by Insertional Mutagenesis. Mol Plant-Microbe Interact 12: 129-142.

47. Segmuller N, Kokkelink L, Giesbert S, Odinius D, van Kan J, et al. (2008) NADPH oxidases are involved in differentiation and pathogenicity in Botrytis cinerea. Mol Plant Microbe Interact 21: 808-819.

48. Spiers AG, Hopcroft DH (1983) Ultrastructural study of the pathogenesis of Marssonina species to poplars. European journal of forest pathology 13: 414-427.

49. van Kan JA, van't Klooster JW, Wagemakers CA, Dees DC, van der Vlugt-Bergmans CJ (1997) Cutinase A of Botrytis cinerea is expressed, but not essential, during penetration of gerbera and tomato. Mol Plant Microbe Interact 10: 30-38.

50. Huertas-Gonzalez MD, Ruiz-Roldan MC, Garcia Maceira FI, Roncero MI, Di Pietro A (1999) Cloning and characterization of pl1 encoding an in planta-secreted pectate lyase of Fusarium oxysporum. Curr Genet 35: 36-40.

51. Hong J, Tamaki H, Yamamoto K, Kumagai H (2003) Cloning of a gene encoding thermostable cellobiohydrolase from Thermoascus aurantiacus and its expression in yeast. Appl Microbiol Biotechnol 63: 42-50.

52. Cheng Q, Cao Y, Jiang C, Xu L, Wang M, et al. (2010) Identifying secreted proteins of Marssonina brunnea by degenerate PCR. Proteomics 10: 2406-2417.

53. Kovalchuk A, Driessen AJ (2010) Phylogenetic analysis of fungal ABC transporters. BMC Genomics 11: 177.

54. Urban M, Bhargava T, Hamer JE (1999) An ATP-driven efflux pump is a novel pathogenicity factor in rice blast disease. EMBO J 18: 512-521.

55. Sun CB, Suresh A, Deng YZ, Naqvi NI (2006) A multidrug resistance transporter in Magnaporthe is required for host penetration and for survival during oxidative stress. Plant Cell 18: 3686-3705.

56. Stefanato FL, Abou-Mansour E, Buchala A, Kretschmer M, Mosbach A, et al. (2009) The ABC transporter BcatrB from Botrytis cinerea exports camalexin and is a virulence factor on Arabidopsis thaliana. Plant J 58: 499-510.

57. Osbourn A, Bowyer P, Lunness P, Clarke B, Daniels M (1995) Fungal pathogens of oat roots and tomato leaves employ closely related enzymes to detoxify different host plant saponins. Mol Plant Microbe Interact 8: 971-978.

58. Pareja-Jaime Y, Roncero MI, Ruiz-Roldan MC (2008) Tomatinase from Fusarium oxysporum f. sp. lycopersici is required for full virulence on tomato plants. Mol Plant Microbe Interact 21: 728-736.

59. Ohsato S, Ochiai-Fukuda T, Nishiuchi T, Takahashi-Ando N, Koizumi S, et al. (2007) Transgenic rice plants expressing trichothecene 3-O-acetyltransferase show resistance to the Fusarium phytotoxin deoxynivalenol. Plant Cell Rep 26: 531-538.

60. Daub ME (1982) Peroxidation of tobacco membrane lipids by the photosensitizing toxin, cercosporin. Plant Physiol 69: 1361-1364.

61. Choquer M, Dekkers KL, Chen HQ, Cao L, Ueng PP, et al. (2005) The CTB1 gene encoding a fungal polyketide synthase is required for cercosporin biosynthesis and fungal virulence of Cercospora nicotianae. Mol Plant Microbe Interact 18: 468-476.

62. Callahan TM, Rose MS, Meade MJ, Ehrenshaft M, Upchurch RG (1999) CFP, the putative cercosporin transporter of Cercospora kikuchii, is required for wild type cercosporin production, resistance, and virulence on soybean. Mol Plant Microbe Interact 12: 901-910.

63. Shim WB, Dunkle LD (2003) CZK3, a MAP kinase kinase kinase homolog in Cercospora zeae-maydis, regulates cercosporin biosynthesis, fungal development, and pathogenesis. Mol Plant Microbe Interact 16: 760-768.

64. Panaccione DG, Scott-Craig JS, Pocard JA, Walton JD (1992) A cyclic peptide synthetase gene required for pathogenicity of the fungus Cochliobolus carbonum on maize. Proc Natl Acad Sci U S A 89: 6590-6594.

65. Tanaka A, Shiotani H, Yamamoto M, Tsuge T (1999) Insertional mutagenesis and cloning of the genes required for biosynthesis of the host-specific AK-toxin in the Japanese pear pathotype of Alternaria alternata. Mol Plant Microbe Interact 12: 691-702.

66. Johnson RD, Johnson L, Itoh Y, Kodama M, Otani H, et al. (2000) Cloning and characterization of a cyclic peptide synthetase gene from Alternaria alternata apple pathotype whose product is involved in AM-toxin synthesis and pathogenicity. Mol Plant Microbe Interact 13: 742-753.

67. Ajiro N, Miyamoto Y, Masunaka A, Tsuge T, Yamamoto M, et al. (2010) Role of the host-selective ACT-toxin synthesis gene ACTTS2 encoding an enoyl-reductase in pathogenicity of the tangerine pathotype of Alternaria alternata. Phytopathology 100: 120-126.

68. Miyamoto Y, Masunaka A, Tsuge T, Yamamoto M, Ohtani K, et al. (2010) ACTTS3 encoding a polyketide synthase is essential for the biosynthesis of ACT-toxin and pathogenicity in the tangerine pathotype of Alternaria alternata. Mol Plant Microbe Interact 23: 406-414.

69. Kahmann R, Basse C (2001) Fungal gene expression during pathogenesis-related development and host plant colonization. Curr Opin Microbiol 4: 374-380.

70. Wahl R, Wippel K, Goos S, Kamper J, Sauer N (2010) A novel high-affinity sucrose transporter is required for virulence of the plant pathogen Ustilago maydis. PLoS Biol 8: e1000303.

71. Eichhorn H, Lessing F, Winterberg B, Schirawski J, Kamper J, et al. (2006) A ferroxidation/permeation iron uptake system is required for virulence in Ustilago maydis. Plant Cell 18: 3332-3345.

72. Voisard C, Wang J, McEvoy JL, Xu P, Leong SA (1993) urbs1, a gene regulating siderophore biosynthesis in Ustilago maydis, encodes a protein similar to the erythroid transcription factor GATA-1. Mol Cell Biol 13: 7091-7100.

73. Hahn M, Neef U, Struck C, Gottfert M, Mendgen K (1997) A putative amino acid transporter is specifically expressed in haustoria of the rust fungus Uromyces fabae. Mol Plant Microbe Interact 10: 438-445.

74. van den Bosch H, Schutgens RB, Wanders RJ, Tager JM (1992) Biochemistry of peroxisomes. Annu Rev Biochem 61: 157-197.

75. Imazaki A, Tanaka A, Harimoto Y, Yamamoto M, Akimitsu K, et al. (2010) Contribution of peroxisomes to secondary metabolism and pathogenicity in the fungal plant pathogen Alternaria alternata. Eukaryot Cell 9: 682-694.

76. Kimura A, Takano Y, Furusawa I, Okuno T (2001) Peroxisomal metabolic function is required for appressorium-mediated plant infection by Colletotrichum lagenarium. Plant Cell 13: 1945-1957.

77. Asakura M, Okuno T, Takano Y (2006) Multiple contributions of peroxisomal metabolic function to fungal pathogenicity in Colletotrichum lagenarium. Appl Environ Microbiol 72: 6345-6354.

78. Lum G, Min XJ (2011) FunSecKB: the Fungal Secretome KnowledgeBase. Database (Oxford) 2011: bar001.

79. Rep M (2005) Small proteins of plant-pathogenic fungi secreted during host colonization. FEMS Microbiol Lett 253: 19-27.

80. Dean RA, Talbot NJ, Ebbole DJ, Farman ML, Mitchell TK, et al. (2005) The genome sequence of the rice blast fungus Magnaporthe grisea. Nature 434: 980-986.

81. Kamper J, Kahmann R, Bolker M, Ma LJ, Brefort T, et al. (2006) Insights from the genome of the biotrophic fungal plant pathogen Ustilago maydis. Nature 444: 97-101.

82. Spanu PD, Abbott JC, Amselem J, Burgis TA, Soanes DM, et al. (2010) Genome expansion and gene loss in powdery mildew fungi reveal tradeoffs in extreme parasitism. Science 330: 1543-1546.

83. Choi J, Park J, Kim D, Jung K, Kang S, et al. (2010) Fungal secretome database: integrated platform for annotation of fungal secretomes. BMC Genomics 11: 105.

84. Tjalsma H, Bolhuis A, Jongbloed JD, Bron S, van Dijl JM (2000) Signal peptide-dependent protein transport in Bacillus subtilis: a genome-based survey of the secretome. Microbiol Mol Biol Rev 64: 515-547.

85. Moller S, Croning MD, Apweiler R (2001) Evaluation of methods for the prediction of membrane spanning regions. Bioinformatics 17: 646-653.

86. van der Vlugt-Bergmans CJ, Wagemakers CA, van Kan JA (1997) Cloning and expression of the cutinase A gene of Botrytis cinerea. Mol Plant Microbe Interact 10: 21-29.

87. Charkowski AO, Alfano JR, Preston G, Yuan J, He SY, et al. (1998) The Pseudomonas syringae pv. tomato HrpW protein has domains similar to harpins and pectate lyases and can elicit the plant hypersensitive response and bind to pectate. J Bacteriol 180: 5211-5217.

88. Yoder MD, Keen NT, Jurnak F (1993) New domain motif: the structure of pectate lyase C, a secreted plant virulence factor. Science 260: 1503-1507.

89. Yakoby N, Beno-Moualem D, Keen NT, Dinoor A, Pines O, et al. (2001) Colletotrichum gloeosporioides pelB is an important virulence factor in avocado fruit-fungus interaction. Mol Plant Microbe Interact 14: 988-995.

90. Marin-Rodriguez MC, Orchard J, Seymour GB (2002) Pectate lyases, cell wall degradation and fruit softening. J Exp Bot 53: 2115-2119.

91. Agrawal RK, Penczek P, Grassucci RA, Frank J (1998) Visualization of elongation factor G on the Escherichia coli 70S ribosome: the mechanism of translocation. Proc Natl Acad Sci U S A 95: 6134-6138.

92. Gacser A, Trofa D, Schafer W, Nosanchuk JD (2007) Targeted gene deletion in Candida parapsilosis demonstrates the role of secreted lipase in virulence. J Clin Invest 117: 3049-3058.

93. Gacser A, Stehr F, Kroger C, Kredics L, Schafer W, et al. (2007) Lipase 8 affects the pathogenesis of Candida albicans. Infect Immun 75: 4710-4718.

94. Voigt CA, Schafer W, Salomon S (2005) A secreted lipase of Fusarium graminearum is a virulence factor required for infection of cereals. Plant J 42: 364-375.

95. Bolton MD, van Esse HP, Vossen JH, de Jonge R, Stergiopoulos I, et al. (2008) The novel Cladosporium fulvum lysin motif effector Ecp6 is a virulence factor with orthologues in other fungal species. Mol Microbiol 69: 119-136.

96. Wan J, Zhang XC, Neece D, Ramonell KM, Clough S, et al. (2008) A LysM receptor-like kinase plays a critical role in chitin signaling and fungal resistance in Arabidopsis. Plant Cell 20: 471-481.

97. Iizasa E, Mitsutomi M, Nagano Y (2010) Direct binding of a plant LysM receptor-like kinase, LysM RLK1/CERK1, to chitin in vitro. J Biol Chem 285: 2996-3004.

98. Zhang XC, Cannon SB, Stacey G (2009) Evolutionary genomics of LysM genes in land plants. BMC Evol Biol 9: 183.

99. Buist G, Steen A, Kok J, Kuipers OP (2008) LysM, a widely distributed protein motif for binding to (peptido)glycans. Mol Microbiol 68: 838-847.

100. Jones JD, Dangl JL (2006) The plant immune system. Nature 444: 323-329.

101. de Jonge R, Bolton MD, Thomma BP (2011) How filamentous pathogens co-opt plants: the ins and outs of fungal effectors. Curr Opin Plant Biol.

102. Stergiopoulos I, de Wit PJ (2009) Fungal effector proteins. Annu Rev Phytopathol 47: 233-263.

103. Guttman DS, Vinatzer BA, Sarkar SF, Ranall MV, Kettler G, et al. (2002) A functional screen for the type III (Hrp) secretome of the plant pathogen Pseudomonas syringae. Science 295: 1722-1726.

104. Jelenska J, Yao N, Vinatzer BA, Wright CM, Brodsky JL, et al. (2007) A J domain virulence effector of Pseudomonas syringae remodels host chloroplasts and suppresses defenses. Curr Biol 17: 499-508.

105. Debuchy R, Berteaux-Lecellier V, Silar P (2010) Mating systems and sexual morphogenesis in Ascomycetes. In: Borkovich KA, Ebbole DJ, editors. Cellular and Molecular Biology of Filamentous Fungi. Washington, DC: ASM Press.

106. Rydholm C, Dyer PS, Lutzoni F (2007) DNA sequence characterization and molecular evolution of MAT1 and MAT2 mating-type loci of the self-compatible ascomycete mold Neosartorya fischeri. Eukaryot Cell 6: 868-874.

107. Faretra F, Antonacci E, Pollastro S (1988) Sexual Behaviour and Mating System of Botryotinia fuckeriana,Teleomorph of Botrytis cinerea. Journal of General Microbiology 134: 2543-2550.

108. Butler G, Kenny C, Fagan A, Kurischko C, Gaillardin C, et al. (2004) Evolution of the MAT locus and its Ho endonuclease in yeast species. Proc Natl Acad Sci U S A 101: 1632-1637.

109. Butler G, Rasmussen MD, Lin MF, Santos MA, Sakthikumar S, et al. (2009) Evolution of pathogenicity and sexual reproduction in eight Candida genomes. Nature 459: 657-662.

110. Woltering D, Baumgartner B, Bagchi S, Larkin B, Loidl J, et al. (2000) Meiotic segregation, synapsis, and recombination checkpoint functions require physical interaction between the chromosomal proteins Red1p and Hop1p. Mol Cell Biol 20: 6646-6658.

111. Hollingsworth NM, Ponte L (1997) Genetic interactions between HOP1, RED1 and MEK1 suggest that MEK1 regulates assembly of axial element components during meiosis in the yeast Saccharomyces cerevisiae. Genetics 147: 33-42.

112. Christensen PU, Davey J, Nielsen O (1997) The Schizosaccharomyces pombe mam1 gene encodes an ABC transporter mediating secretion of M-factor. Mol Gen Genet 255: 226-236.

113. Oelschlaegel T, Schwickart M, Matos J, Bogdanova A, Camasses A, et al. (2005) The yeast APC/C subunit Mnd2 prevents premature sister chromatid separation triggered by the meiosis-specific APC/C-Ama1. Cell 120: 773-788.

114. Agarwal S, Roeder GS (2000) Zip3 provides a link between recombination enzymes and synaptonemal complex proteins. Cell 102: 245-255.

115. Lorenz A, Wells JL, Pryce DW, Novatchkova M, Eisenhaber F, et al. (2004) S. pombe meiotic linear elements contain proteins related to synaptonemal complex components. J Cell Sci 117: 3343-3351.

116. Loidl J (2006) S. pombe linear elements: the modest cousins of synaptonemal complexes. Chromosoma 115: 260-271.

117. Molnar M, Doll E, Yamamoto A, Hiraoka Y, Kohli J (2003) Linear element formation and their role in meiotic sister chromatid cohesion and chromosome pairing. J Cell Sci 116: 1719-1731.

118. Niu H, Wan L, Baumgartner B, Schaefer D, Loidl J, et al. (2005) Partner choice during meiosis is regulated by Hop1-promoted dimerization of Mek1. Mol Biol Cell 16: 5804-5818.

119. Tsubouchi H, Roeder GS (2004) The budding yeast mei5 and sae3 proteins act together with dmc1 during meiotic recombination. Genetics 168: 1219-1230.

120. Celerin M, Merino ST, Stone JE, Menzie AM, Zolan ME (2000) Multiple roles of Spo11 in meiotic chromosome behavior. EMBO J 19: 2739-2750.

121. Benjamin KR, Zhang C, Shokat KM, Herskowitz I (2003) Control of landmark events in meiosis by the CDK Cdc28 and the meiosis-specific kinase Ime2. Genes Dev 17: 1524-1539.

122. Liang F, Jin F, Liu H, Wang Y (2009) The molecular function of the yeast polo-like kinase Cdc5 in Cdc14 release during early anaphase. Mol Biol Cell 20: 3671-3679.

**Supplementary Figure Legends**

**Figure S1** The structure of ITS (internal transcribed spacer) DNA sequence. ITS1 was located between the SSU (small subunit) RNA and 5.8s RNA, and ITS2 was located between the 5.8s RNA and LSU (large subunit) RNA.

**Figure S2** The taxonomic classification of three fungi including *M. brunnea*, *B. cinerea* and *S. sclerotiorum*.

**Figure S3** The distribution of protein families in *M. brunnea*.

**Figure S4. Pathogen protection mechanism during infection.** Fungi have mechanisms to avoid induction of the host immunity systems and alleviate the defense responses. The fungal plant pathogen *C. fulvum* gene ECP6 encodes a small, secreted protein, which sequesters chitin oligosaccharides to prevent eliciting host defense responses. Pathogens have two methods of coping with the toxicity and antifungal compound secreted by the host. One is efflux by the ABC1-encoded protein. The other is to produce enzymes to degrade them: *Gaeumannomyces graminis* secrets saponin-degrading enzymes AVENACINASE to detoxify the triterpenoid oat root saponin avenacin A-1. As the pathogens can secret some substances that contribute to infection that are also harmful to the pathogen itself, pathogen should encode methods of mitigating self-harm. *Fusarium sporotrichioides* can produce the trichothecene mycotoxin deoxynivalenol (DON) to inhibit protein synthesis of the host. The fungi have a gene called *TRI101* that encodes trichothecene 3-O acetyltransferase, which can reduce the damage to pathogen caused by trichothecene mycotoxin deoxynivalenol.

**Figure S5** The domain structure for the gene *ABC3*.

**Figure S6. Fungal toxin biosynthesis.** Fungi produce toxins to destroy host cellular functions. They can be non-host specific or host specific. Fungi have many genes to control the biosynthesis, export, and regulation of the toxins. Cercosporin is a non-host specific toxin. A polyketide synthase gene, CTB1, plays a key role in cercosporin biosynthesis. CFP encodes a cercosporin Transporter exporting cercosporin, CZK3, which regulates cercosporin biosynthesis. Comparing to the non-host specific toxins, some toxins are active only toward hosts, i.e. host specific toxins, such as HC-toxin, AK-toxin, AM-toxin, and ACT-toxin. HTS1 encodes a multifunctional cyclic peptide synthetase involved in the biosynthesis of HC-toxin. Besides HTS1, ToxC and ToxF are also essential for toxin biosynthesis and pathogenicity. AKT1, which encodes a series of carboxyl-activating enzymes, and AKT2 are involved in the biosynthesis of the AK-toxin. The AMT gene is essential for the biosynthesis of the AM-toxin. ACTTS2 and ACTTS3 are essential genes for ACT-toxin biosynthesis.

**Figure S7. Multiple alignment of 28 putative proteins with highly similarity for *M. brunnea*.** Multiple sequence alignment of the 28 putative proteins was performed using ClustalW.

Figure S1


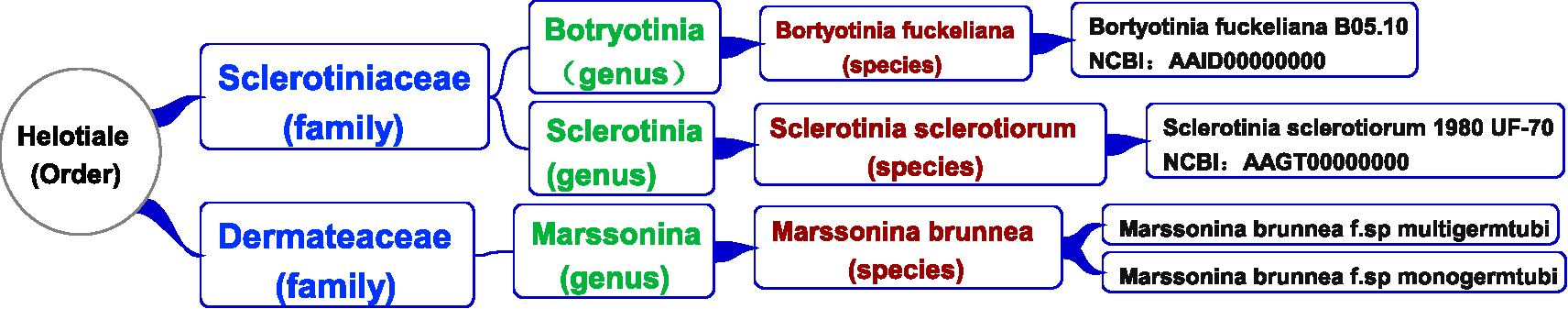


Figure S2

Figure S3

Figure S4

Figure S5


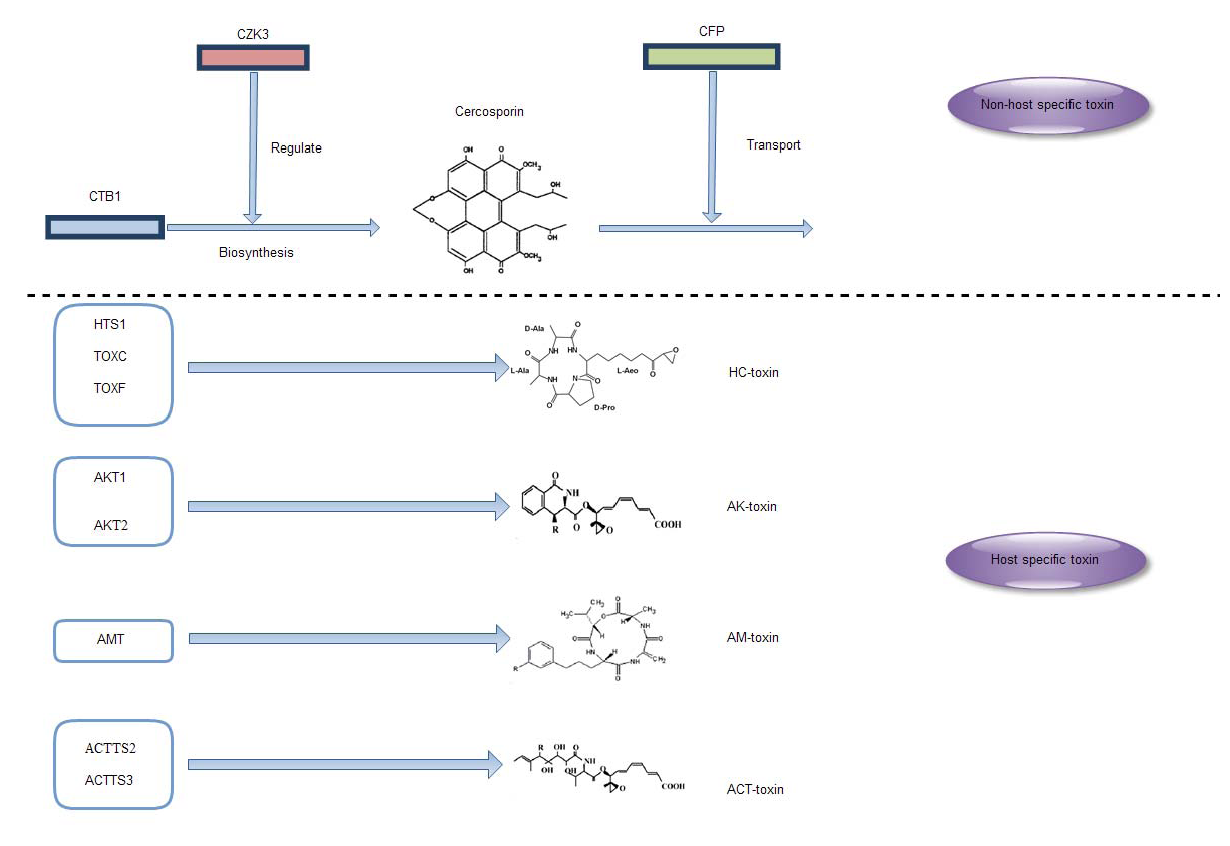


Figure S6

Figure S7

Table S1 Main features of *M. brunnea* genome assemblies.

|  | Non-gap Closure | | Gap Closure | |
| --- | --- | --- | --- | --- |
|  | Contig | Scaffold | Contig | Scaffold |
| No. of contig/scaffold | 2,990 | 155 | 2,420 | 90 |
| N50 size of contig/scaffold | 33,873 | 1,359,333 | 39,335 | 1,599,552 |
| Mean length of contig/scaffold | 17,565 | 340,660 | 21,398 | 577,958 |
| Minimum length of contig/scaffold | 1,002 | 1,006 | 1,002 | 1,006 |
| Maximum length of contig/scaffold | 235,302 | 3,606,805 | 246,791 | 5,185,404 |
| Genome Size | 52.5Mbp | 52.8Mbp | 51.78Mbp | 52Mbp |
| GC-content (%) | 42.69% | 42.46% | 42.90% | 42.71% |

N50 is widely used for measuring the average length of assembled sequences in genome assembly and is defined as the largest length N for which the sum of the sequences that are more than or equal the length N are not less than half of the genome sizes.

| Table S2 | The number of tRNAs in the mtDNA. |
| --- | --- |

|  | Ile | Arg | Leu | Lys | Phe | Sec | Thr | Tyr | Cys |
| --- | --- | --- | --- | --- | --- | --- | --- | --- | --- |
| Number | 5 | 4 | 2 | 2 | 2 | 2 | 2 | 2 | 1 |

Table S3 The number of RNA-seq reads mapped to the genome of *Populus* and *M. brunnea.*

| Simple | Genomic Type | Total | | Unique | |
| --- | --- | --- | --- | --- | --- |
|  |  | Number | Percent (%) | Number | Percent (%) |
| M6 | *M. brunnea* | 13,805,307 | 63.7 | 13,789,461 | 63.63 |
| 895 | Poplar | 22,968,562 | 85.34 | 13,962,858 | 51.88 |
| 895-M6 | *M. brunnea* | 1,014,034 | 4.12 | 1,009,666 | 4.11 |
|  | Poplar | 20,375,687 | 82.84 | 11,063,080 | 44.98 |
|  | Total | 21,389,721 | 86.96 | 12,072,746 | 49.09 |

| Sample M6: *M. brunnea* spores collected from potato dextrose agor. | | |  | |  |
| --- | --- | --- | --- | --- | --- |
| Sample 895-M6: the leaves of poplar (NL895) after 96 hours of infection by *M. brunnea.* | | | | | |
| Sample 895: the leaves of poplar (NL895). |  |  | |  |  |

Table S4 Protein families with more than 10 genes that were up-regulated in *M. brunnea*.

| Pfam Id | Family Name | Number | | |
| --- | --- | --- | --- | --- |
|  |  | total | up-regulation | down-regulation |
| PF01476 | LysM domain | 33 | 30 | 0 |
| PF07690 | Major Facilitator Superfamily | 134 | 24 | 15 |
| PF04082 | Fungal specific transcription factor domain | 58 | 21 | 3 |
| PF00172 | Fungal Zn(2)-Cys(6) binuclear cluster domain | 52 | 17 | 3 |
| PF00400 | WD domain, G-beta repeat | 86 | 16 | 3 |
| PF00069 | Protein kinase domain | 105 | 15 | 4 |
| PF00083 | Sugar (and other) transporter | 47 | 14 | 5 |
| PF00106 | Short chain dehydrogenase | 73 | 12 | 13 |
| PF00176 | SNF2 family N-terminal domain | 25 | 11 | 0 |
| PF00501 | AMP-binding enzyme | 26 | 11 | 0 |
| PF00122 | E1-E2 ATPase | 19 | 10 | 1 |
| PF00657 | GDSL-like Lipase/Acylhydrolase | 15 | 10 | 0 |

| Table S5 | Resistance genes (R) with differential expression in *Populus*. |
| --- | --- |

|  |  |  |  | Prg (Plant Resistance Genes db) |  |
| --- | --- | --- | --- | --- | --- |
| JGI_ID | Type | Ratio | Prg_ID | Organism | Name |
| 590083 | up | 524 | 47388 | Populus trichocarpa | NBS-LRR type R-gene |
| 757233 | up | 198 | 47382 | Populus trichocarpa | NBS-LRR type R-gene |
| 791717 | up | 139 | 47389 | Populus trichocarpa | NBS-LRR type R-gene |
| 757234 | up | 100 | 47390 | Populus trichocarpa | NBS-LRR type R-gene |
| 264262 | up | 13 | 49045 | Populus alba | LRR type R-gene(lrr1) |
| 583412 | up | 13 | 47390 | Populus trichocarpa | NBS-LRR type R-gene |
| 783621 | up | 11 | 47374 | Populus trichocarpa | NBS type R-gene |
| 590084 | up | 10 | 47390 | Populus trichocarpa | NBS-LRR type R-gene |
| 590077 | up | 10 | 47390 | Populus trichocarpa | NBS-LRR type R-gene |
| 819526 | up | 9 | 51457 | Arabidopsis thaliana | histone serine kinase |
| 264245 | up | 6 | 50593 | Populus trichocarpa | TIR-NBS type R-gene |
| 819198 | up | 5 | 51314 | Arabidopsis thaliana | ATP binding |
| 813757 | up | 5 | 51100 | Aquilegia | Putative R-Genes (EST1172288) |
| 731636 | up | 5 | 51471 | Arabidopsis thaliana | shaggy-like kinase 13 |
| 583893 | up | 5 | 47405 | Populus trichocarpa | TIR-NBS-LRR-TIR type R-gene |
| 278686 | up | 3 | 47375 | Populus trichocarpa | NBS type R-gene |
| 792108 | up | 3 | 49800 | Populus tremula | NBS-LRR type R-gene |
| 788329 | up | 3 | 50617 | Populus tremula | P1-RGA10 R-gene |
| 816604 | up | 2 | 51663 | Arabidopsis thaliana | ATMPK12(MAP kinase) |
| 410239 | up | 2 | 51290 | Arabidopsis thaliana | BR-signaling kinase 1 |
| 829617 | down | 0.04 | 51314 | Arabidopsis thaliana | ATP binding |
| 199158 | down | 0.04 | 51553 | Arabidopsis thaliana | NIK1 |
| 266031 | down | 0.12 | 51553 | Arabidopsis thaliana | NIK1 |
| 717990 | down | 0.15 | 51156 | Arabidopsis thaliana | BAM2 |
| 576722 | down | 0.16 | 47365 | Populus trichocarpa | NBS-LRR type R-gene |
| 835158 | down | 0.21 | 51513 | Arabidopsis thaliana | Protein kinase superfamily |
| 815301 | down | 0.41 | 481 | Cucumis melo | aminotransferase |
| 226191 | down | 0.46 | 47297 | Brassica rapa | LRR-like R-gene |
| 254607 | down | 0.49 | 51352 | Arabidopsis thaliana | Protein kinase family |
| 822933 | down | 0.55 | 51359 | Arabidopsis thaliana | BIN2 |
| 207656 | down | 0.59 | 51221 | Arabidopsis thaliana | SERK2 |
| 723016 | down | 0.6 | 481 | Cucumis melo | aminotransferase |
| 650389 | down | 0.7 | 51001 | Aquilegia | EST1128655 |
| 282541 | down | 0.76 | 51352 | Arabidopsis thaliana | protein kinase family |
| 581623 | down | 0.79 | 51471 | Arabidopsis thaliana | protein kinase family |
| 672762 | down | 0.81 | 51310 | Arabidopsis thaliana | protein kinase family |

Table S6 The GenBank accession no of ITS sequences used for phylogenetic tree analysis.

| Species | Strain | Accession no |
| --- | --- | --- |
| *Botrytis cinerea* | BC_1283 | EF207415 |
| *Botrytis cinerea* | BC_BC12 | GU724512 |
| *Botrytis cinerea* | BC_FSU6300 | GQ221113 |
| *Sclerotinia_sclerotiorum* | SS_ms82 | HQ833447 |
| *Sclerotinia_sclerotiorum* | SS_ms83 | HQ833448 |
| *Sclerotinia_sclerotiorum* | SS_ms84 | HQ833449 |
| *Marssonina coronariae* | MC_KR-AP-1 | GQ456166 |
| *Marssonina coronariae* | MC_LLH-M8-12 | HM368521 |
| *Marssonina coronariae* | MC_LLHs-196 | HM368520 |
| *Marssonina coronariae* | MC_ZXR-YL-Ye-1 | EU329735 |
| *Marssonina coronariae* | MC_ZXR-YL-Ye-2 | EU329734 |
| *Marssonina coronariae* | MC_ZXR-YL-Ye-3 | EU329732 |
| *Marssonina coronariae* | MC_ZXR-bshb-1 | EU329733 |
| *Marssonina coronariae* | MC_lk3 | FJ606802 |
| *Marssonina coronariae* | MC_lsl | FJ606800 |
| *Marssonina coronariae* | MC_wg5l | FJ606799 |
| *Marssonina coronariae* | MC_wgl | FJ606798 |
| *Marssonina coronariae* | MC_zgh | FJ606801 |
| *Marssonina rosae* | MR_26L-435-Mexico | AY904059 |
| *Marssonina rosae* | MR_RM071209004 | FJ493247 |
| *Marssonina rosae* | MR_RM071209006 | FJ493242 |
| *Marssonina rosae* | MR_RM080715007 | FJ493248 |
| *Marssonina rosae* | MR_RM080715008 | FJ493249 |
| *Marssonina rosae* | MR_UASWS0428 | HM235978 |

Table S7 The distribution of low complexity sequences for *M. brunnea, B. cinerea, and S. sclerotiorum*.

| Type | *M. brunnea* | | *B. cinerea* | | *S. sclerotiorum* | |
| --- | --- | --- | --- | --- | --- | --- |
|  | Number | Length | Number | Length | Number | Length |
| A-rich | 508 | 36,464 | 232 | 16,050 | 272 | 19,811 |
| AT-rich | 1,838 | 71,465 | 2,090 | 148,353 | 3,454 | 237,945 |
| C-rich | 449 | 31,654 | 164 | 13,180 | 198 | 18,092 |
| CT-rich | 501 | 37,689 | 288 | 23,211 | 261 | 23,619 |
| G-rich | 476 | 34021 | 155 | 13458 | 208 | 17550 |
| GA-rich | 472 | 35,602 | 294 | 23,954 | 302 | 24,686 |
| GC_rich | 116 | 3,186 | 17 | 594 | 5 | 109 |
| T-rich | 486 | 35,038 | 215 | 14,707 | 288 | 20,899 |
| polypurine | 28 | 1,347 | 8 | 452 | 11 | 785 |
| polypyrimidine | 25 | 1,092 | 9 | 595 | 7 | 443 |
| Total | 4,899 | 287,558 | 3,472 | 254,554 | 5,006 | 363,939 |

Table S8. The distribution of simple repeat sequences for *M. brunnea, B. cinerea, and S. sclerotiorum*.

| Unit Size | *M. brunnea* | | *B. cinerea* | | *S. sclerotiorum* | |
| --- | --- | --- | --- | --- | --- | --- |
|  | Number | Length | Number | Length | Number | Length |
| 1 | 1,057 | 35,036 | 256 | 7,554 | 197 | 5,860 |
| 2 | 1,996 | 82,222 | 736 | 28,715 | 270 | 11,498 |
| 3 | 3,335 | 146,412 | 1,027 | 48,864 | 733 | 37,879 |
| 4 | 4,038 | 180,441 | 2,256 | 93,078 | 1,988 | 82,799 |
| 5 | 3,676 | 163,598 | 2,301 | 88,356 | 1,919 | 80,841 |
| 6 | 932 | 46,214 | 483 | 22,242 | 386 | 19,805 |
| Total | 15,034 | 653,923 | 7,059 | 288,809 | 5,493 | 238,682 |

Table S9 Top 20 protein families in *M. brunnea* that are the most significantly different from those of other fungal genomes including *B. cinerea, S. sclerotiorum, M. grisea, and F. graminearum.*

| Pfam ID | Family Name | *M. brunnea* | *B. cinerea* | *S. sclerotiorum* | *M. grisea* | *F.* | *p*-value |
| --- | --- | --- | --- | --- | --- | --- | --- |
|  |  |  |  |  |  | *graminearum* |  |
| PF01476 | LysM domain | 33 | 4 | 6 | 7 | 8 | 7.15E-05 |
| PF01408 | Oxidoreductase family, NAD-binding Rossmann fold | 8 | 2 | 2 | 1 | 2 | 0.00014 |
| TIGR02169 | SMC_prok_A: chromosome segregation protein SMC | 6 | 0 | 0 | 0 | 1 | 0.00018 |
| PF00076 | RNA recognition motif. (a.k.a. RRM, RBD, or RNP domain) | 54 | 38 | 34 | 35 | 37 | 0.000285 |
| TIGR02168 | SMC_prok_B: chromosome segregation protein SMC | 14 | 0 | 1 | 2 | 3 | 0.000301 |
| PF00125 | Core histone H2A/H2B/H3/H4 | 16 | 10 | 8 | 8 | 9 | 0.000625 |
| PF00534 | Glycosyl transferases group 1 | 8 | 5 | 4 | 4 | 4 | 0.000643 |
| PF03446 | NAD binding domain of 6-phosphogluconate dehydrogenase | 7 | 2 | 2 | 3 | 1 | 0.001172 |
| PF01753 | MYND finger | 17 | 8 | 4 | 7 | 7 | 0.001208 |
| PF00004 | ATPase family associated with various cellular activities (AAA) | 26 | 4 | 4 | 10 | 9 | 0.001237 |
| PF01757 | Acyltransferase family | 10 | 2 | 5 | 4 | 3 | 0.002085 |
| PF03169 | OPT oligopeptide transporter protein | 7 | 12 | 12 | 12 | 11 | 0.000318 |
| PF00005 | ABC transporter | 11 | 25 | 22 | 22 | 21 | 0.000923 |
| PF04909 | Amidohydrolase | 4 | 8 | 7 | 7 | 8 | 0.001208 |
| PF00722 | Glycosyl hydrolases family 16 | 10 | 20 | 18 | 17 | 20 | 0.001353 |
| PF00664 | ABC transporter transmembrane region | 12 | 18 | 17 | 18 | 20 | 0.00217 |
| PF00067 | Cytochrome P450 | 50 | 127 | 94 | 131 | 112 | 0.004289 |
| PF07992 | Pyridine nucleotide-disulphide oxidoreductase | 18 | 26 | 24 | 22 | 26 | 0.006533 |
| PF00698 | Acyl transferase domain | 2 | 6 | 5 | 6 | 4 | 0.006533 |
| PF01494 | FAD binding domain | 22 | 45 | 33 | 38 | 44 | 0.007621 |

Table S10 Six gene groups involved in pathogenesis

Table S11 The genes associated with mating and meiosis.

Table S12 The number of putative secretory proteins among *U. maydis*, *M. grisea*, *B. cinerea*, *S. sclerotiorum*, and *M. brunnea.*

| Organism | Total Number | Secretory Protein | Percent (%) |
| --- | --- | --- | --- |
| *U. maydis* | 6,522 | 431 | 6.61 |
| *M. grisea* | 11,054 | 1471 | 13.31 |
| *B. cinerea* | 16,448 | 755 | 4.59 |
| *S. sclerotiorum* | 14,522 | 623 | 4.29 |
| *M. brunnea* | 10,040 | 599 | 5.97 |

| Total Number: the total number of proteins in a genome |
| --- |
| Secretory Protein: the total number of secreted proteins in a genome |

Table S13 The secretory protein families with more than five members *M. brunnea*.

| Pfam ID | Family Name | Number |
| --- | --- | --- |
| PF01476 | LysM | 29 |
| PF00544 | Pec_lyase_C | 8 |
| PF00657 | GDSL-like Lipase/Acylhydrolase | 8 |
| PF01083 | Cutinase | 8 |
| PF03443 | Glyco_hydro_61 | 8 |
| PF00135 | Carboxylestase family | 6 |
| PF00150 | Cellulase (glycosyl hydrolase family 5) | 6 |
| PF00264 | Common central domain of tyrosinase | 6 |
| PF01565 | FAD binding domain | 6 |
| PF04616 | Glycosyl hydrolases family 43 | 6 |
| PF00732 | GMC oxidoreductase | 5 |
| PF03211 | Pectate lyase | 5 |
| PF05730 | CFEM domain | 5 |
|  |  |  |
| Number: the total number of secreted protein in protein family. | |  |
