# Supplementary material for: Sequencing the genome of Marssonina brunnea reveals fungus-poplar co-evolution
Source: BMC Genomics. 2012 Aug 9;13:382. doi: 10.1186/1471-2164-13-382 (PMC3484023; doi:10.1186/1471-2164-13-382)
Supplement: Additional file 11 — Table S5. The genes associated with mating and meiosis. [file 1471-2164-13-382-S11.doc]

| Table S5 | Resistance genes (R) with differential expression in *Populus*. |
| --- | --- |

|  |  |  |  | Prg (Plant Resistance Genes db) |  |
| --- | --- | --- | --- | --- | --- |
| JGI_ID | Type | Ratio | Prg_ID | Organism | Name |
| 590083 | up | 524 | 47388 | Populus trichocarpa | NBS-LRR type R-gene |
| 757233 | up | 198 | 47382 | Populus trichocarpa | NBS-LRR type R-gene |
| 791717 | up | 139 | 47389 | Populus trichocarpa | NBS-LRR type R-gene |
| 757234 | up | 100 | 47390 | Populus trichocarpa | NBS-LRR type R-gene |
| 264262 | up | 13 | 49045 | Populus alba | LRR type R-gene(lrr1) |
| 583412 | up | 13 | 47390 | Populus trichocarpa | NBS-LRR type R-gene |
| 783621 | up | 11 | 47374 | Populus trichocarpa | NBS type R-gene |
| 590084 | up | 10 | 47390 | Populus trichocarpa | NBS-LRR type R-gene |
| 590077 | up | 10 | 47390 | Populus trichocarpa | NBS-LRR type R-gene |
| 819526 | up | 9 | 51457 | Arabidopsis thaliana | histone serine kinase |
| 264245 | up | 6 | 50593 | Populus trichocarpa | TIR-NBS type R-gene |
| 819198 | up | 5 | 51314 | Arabidopsis thaliana | ATP binding |
| 813757 | up | 5 | 51100 | Aquilegia | Putative R-Genes (EST1172288) |
| 731636 | up | 5 | 51471 | Arabidopsis thaliana | shaggy-like kinase 13 |
| 583893 | up | 5 | 47405 | Populus trichocarpa | TIR-NBS-LRR-TIR type R-gene |
| 278686 | up | 3 | 47375 | Populus trichocarpa | NBS type R-gene |
| 792108 | up | 3 | 49800 | Populus tremula | NBS-LRR type R-gene |
| 788329 | up | 3 | 50617 | Populus tremula | P1-RGA10 R-gene |
| 816604 | up | 2 | 51663 | Arabidopsis thaliana | ATMPK12(MAP kinase) |
| 410239 | up | 2 | 51290 | Arabidopsis thaliana | BR-signaling kinase 1 |
| 829617 | down | 0.04 | 51314 | Arabidopsis thaliana | ATP binding |
| 199158 | down | 0.04 | 51553 | Arabidopsis thaliana | NIK1 |
| 266031 | down | 0.12 | 51553 | Arabidopsis thaliana | NIK1 |
| 717990 | down | 0.15 | 51156 | Arabidopsis thaliana | BAM2 |
| 576722 | down | 0.16 | 47365 | Populus trichocarpa | NBS-LRR type R-gene |
| 835158 | down | 0.21 | 51513 | Arabidopsis thaliana | Protein kinase superfamily |
| 815301 | down | 0.41 | 481 | Cucumis melo | aminotransferase |
| 226191 | down | 0.46 | 47297 | Brassica rapa | LRR-like R-gene |
| 254607 | down | 0.49 | 51352 | Arabidopsis thaliana | Protein kinase family |
| 822933 | down | 0.55 | 51359 | Arabidopsis thaliana | BIN2 |
| 207656 | down | 0.59 | 51221 | Arabidopsis thaliana | SERK2 |
| 723016 | down | 0.6 | 481 | Cucumis melo | aminotransferase |
| 650389 | down | 0.7 | 51001 | Aquilegia | EST1128655 |
| 282541 | down | 0.76 | 51352 | Arabidopsis thaliana | protein kinase family |
| 581623 | down | 0.79 | 51471 | Arabidopsis thaliana | protein kinase family |
| 672762 | down | 0.81 | 51310 | Arabidopsis thaliana | protein kinase family |
