# Supplementary material for: Sequencing the genome of Marssonina brunnea reveals fungus-poplar co-evolution
Source: BMC Genomics. 2012 Aug 9;13:382. doi: 10.1186/1471-2164-13-382 (PMC3484023; doi:10.1186/1471-2164-13-382)
Supplement: Additional file 12 — Table S6. The number of RNA-seq reads mapped to the genome of Populus and M. brunnea. [file 1471-2164-13-382-S12.doc]

Table S3 The number of RNA-seq reads mapped to the genome of *Populus* and *M. brunnea.*

| Simple | Genomic Type | Total | | Unique | |
| --- | --- | --- | --- | --- | --- |
|  |  | Number | Percent (%) | Number | Percent (%) |
| M6 | *M. brunnea* | 13,805,307 | 63.7 | 13,789,461 | 63.63 |
| 895 | Poplar | 22,968,562 | 85.34 | 13,962,858 | 51.88 |
| 895-M6 | *M. brunnea* | 1,014,034 | 4.12 | 1,009,666 | 4.11 |
|  | Poplar | 20,375,687 | 82.84 | 11,063,080 | 44.98 |
|  | Total | 21,389,721 | 86.96 | 12,072,746 | 49.09 |

| Sample M6: *M. brunnea* spores collected from potato dextrose agor. | | |  | |  |
| --- | --- | --- | --- | --- | --- |
| Sample 895-M6: the leaves of poplar (NL895) after 96 hours of infection by *M. brunnea.* | | | | | |
| Sample 895: the leaves of poplar (NL895). |  |  | |  |  |
