# Supplementary material for: Sequencing the genome of Marssonina brunnea reveals fungus-poplar co-evolution
Source: BMC Genomics. 2012 Aug 9;13:382. doi: 10.1186/1471-2164-13-382 (PMC3484023; doi:10.1186/1471-2164-13-382)
Supplement: Additional file 13 — Table S7. Protein families with more than 10 genes that were up-regulated in M. brunnea. [file 1471-2164-13-382-S13.doc]

Table S7 Protein families with more than 10 genes that were up-regulated in *M. brunnea*.

| Pfam Id | Family Name | Number | | |
| --- | --- | --- | --- | --- |
|  |  | total | up-regulation | down-regulation |
| PF01476 | LysM domain | 33 | 30 | 0 |
| PF07690 | Major Facilitator Superfamily | 134 | 24 | 15 |
| PF04082 | Fungal specific transcription factor domain | 58 | 21 | 3 |
| PF00172 | Fungal Zn(2)-Cys(6) binuclear cluster domain | 52 | 17 | 3 |
| PF00400 | WD domain, G-beta repeat | 86 | 16 | 3 |
| PF00069 | Protein kinase domain | 105 | 15 | 4 |
| PF00083 | Sugar (and other) transporter | 47 | 14 | 5 |
| PF00106 | Short chain dehydrogenase | 73 | 12 | 13 |
| PF00176 | SNF2 family N-terminal domain | 25 | 11 | 0 |
| PF00501 | AMP-binding enzyme | 26 | 11 | 0 |
| PF00122 | E1-E2 ATPase | 19 | 10 | 1 |
| PF00657 | GDSL-like Lipase/Acylhydrolase | 15 | 10 | 0 |
