# Supplementary material for: Sequencing the genome of Marssonina brunnea reveals fungus-poplar co-evolution
Source: BMC Genomics. 2012 Aug 9;13:382. doi: 10.1186/1471-2164-13-382 (PMC3484023; doi:10.1186/1471-2164-13-382)
Supplement: Additional file 14 — Figure S7. Multiple alignment of 28 putative proteins with highly similarity for M. brunnea. Multiple sequence alignment of the 28 putative proteins was performed using ClustalW. [file 1471-2164-13-382-S14.pdf]

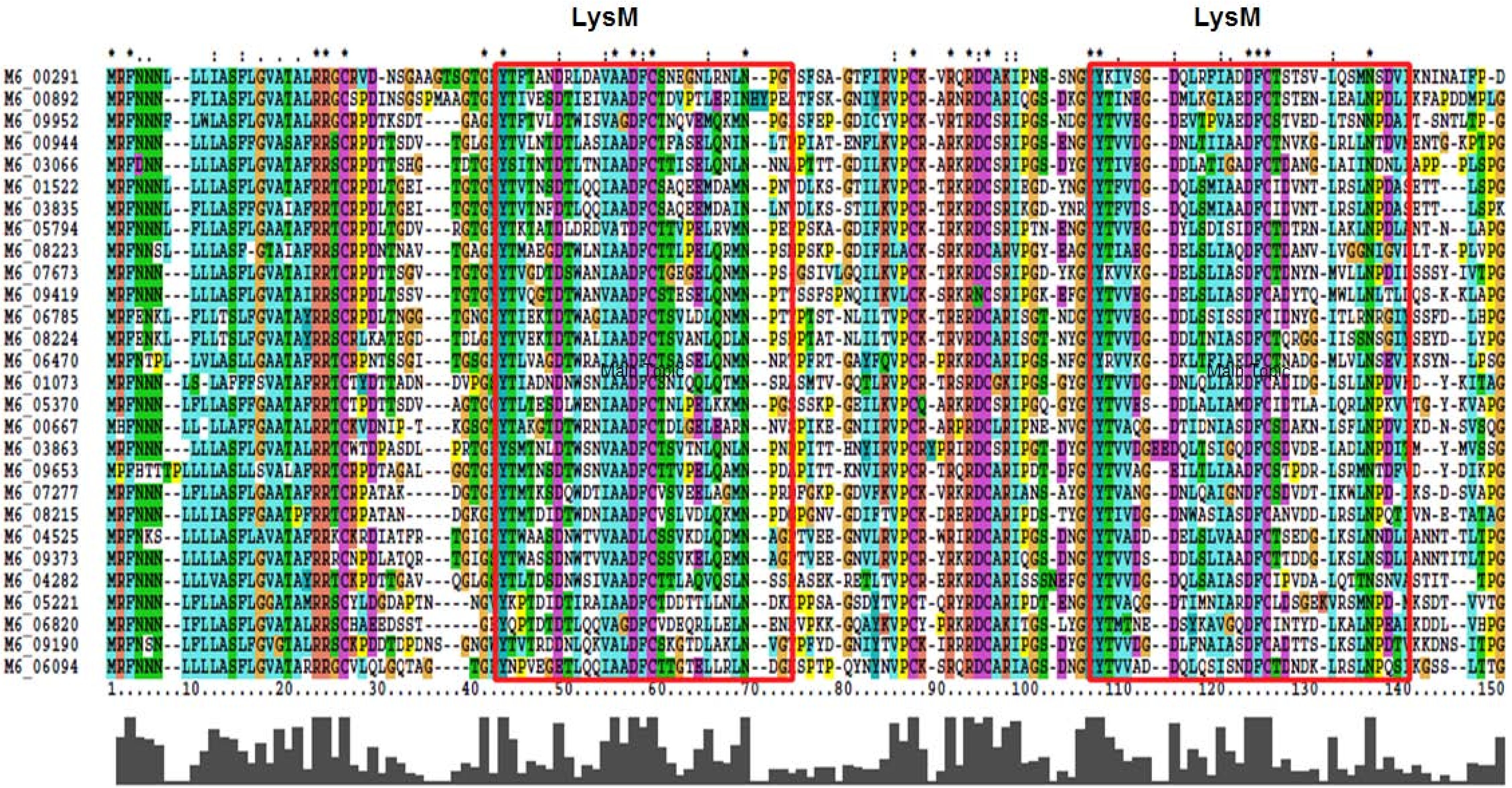
LysM
LysM
M6_00291
M6_00892
M6_09952
M6_00944
M6_03066
M6_01522
M6_03835
M6_05794
M6_08223
M6_07673
M6_09419
M6_06785
M6_08224
M6_06470
M6_01073
M6_05370
M6_00667
M6_03863
M6_09653
M6_07277
M6_08215
M6_04525
M6_09373
M6_04282
M6_05221
M6_06820
M6_09190
M6_06094
