# Supplementary material for: Sequencing the genome of Marssonina brunnea reveals fungus-poplar co-evolution
Source: BMC Genomics. 2012 Aug 9;13:382. doi: 10.1186/1471-2164-13-382 (PMC3484023; doi:10.1186/1471-2164-13-382)
Supplement: Additional file 16 — Table S9. The distribution of low complexity sequences for M. brunnea, B. cinerea, and S. sclerotiorum. [file 1471-2164-13-382-S16.doc]

Table S9 The distribution of low complexity sequences for *M. brunnea, B. cinerea, and S. sclerotiorum*.

| Type | *M. brunnea* | | *B. cinerea* | | *S. sclerotiorum* | |
| --- | --- | --- | --- | --- | --- | --- |
|  | Number | Length | Number | Length | Number | Length |
| A-rich | 508 | 36464 | 232 | 16050 | 272 | 19811 |
| AT-rich | 1838 | 71465 | 2090 | 148353 | 3454 | 237945 |
| C-rich | 449 | 31654 | 164 | 13180 | 198 | 18092 |
| CT-rich | 501 | 37689 | 288 | 23211 | 261 | 23619 |
| G-rich | 476 | 34021 | 155 | 13458 | 208 | 17550 |
| GA-rich | 472 | 35602 | 294 | 23954 | 302 | 24686 |
| GC_rich | 116 | 3186 | 17 | 594 | 5 | 109 |
| T-rich | 486 | 35038 | 215 | 14707 | 288 | 20899 |
| polypurine | 28 | 1347 | 8 | 452 | 11 | 785 |
| polypyrimidine | 25 | 1092 | 9 | 595 | 7 | 443 |
| Total | 4899 | 287558 | 3472 | 254554 | 5006 | 363939 |
