# Supplementary material for: Sequencing the genome of Marssonina brunnea reveals fungus-poplar co-evolution
Source: BMC Genomics. 2012 Aug 9;13:382. doi: 10.1186/1471-2164-13-382 (PMC3484023; doi:10.1186/1471-2164-13-382)
Supplement: Additional file 17 — Table S10. The distribution of simple repeat sequences for M. brunnea, B. cinerea, and S. sclerotiorum. [file 1471-2164-13-382-S17.doc]

Table S10. The distribution of simple repeat sequences for *M. brunnea, B. cinerea, and S. sclerotiorum*.

| Unit Size | *M. brunnea* | | *B. cinerea* | | *S. sclerotiorum* | |
| --- | --- | --- | --- | --- | --- | --- |
|  | Number | Length | Number | Length | Number | Length |
| 1 | 1057 | 35036 | 256 | 7554 | 197 | 5860 |
| 2 | 1996 | 82222 | 736 | 28715 | 270 | 11498 |
| 3 | 3335 | 146412 | 1027 | 48864 | 733 | 37879 |
| 4 | 4038 | 180441 | 2256 | 93078 | 1988 | 82799 |
| 5 | 3676 | 163598 | 2301 | 88356 | 1919 | 80841 |
| 6 | 932 | 46214 | 483 | 22242 | 386 | 19805 |
| Total | 15034 | 653923 | 7059 | 288809 | 5493 | 238682 |
