# Supplementary material for: Sequencing the genome of Marssonina brunnea reveals fungus-poplar co-evolution
Source: BMC Genomics. 2012 Aug 9;13:382. doi: 10.1186/1471-2164-13-382 (PMC3484023; doi:10.1186/1471-2164-13-382)
Supplement: Additional file 18 — Table S11. The number of putative secretory proteins among U. maydis, M. grisea, B. cinerea, S. sclerotiorum, and M. brunnea. [file 1471-2164-13-382-S18.doc]

Table S11 The number of putative secretory proteins among *U. maydis*, *M. grisea*, *B. cinerea*, *S. sclerotiorum*, and *M. brunnea.*

| Organism | Total Number | Secretory Protein | Percent (%) |
| --- | --- | --- | --- |
| *U. maydis* | 6,522 | 431 | 6.61 |
| *M. grisea* | 11,054 | 1471 | 13.31 |
| *B. cinerea* | 16,448 | 755 | 4.59 |
| *S. sclerotiorum* | 14,522 | 623 | 4.29 |
| *M. brunnea* | 10,040 | 599 | 5.97 |

| *Total Number: the total number of proteins in a genome* |
| --- |
| *Secretory Protein: the total number of secreted proteins in a genome* |

Table S12 The secretory protein families with more than five members *M. brunnea*.

| Pfam ID | Family Name | Number |
| --- | --- | --- |
| PF01476 | LysM | 29 |
| PF00544 | Pec_lyase_C | 8 |
| PF00657 | GDSL-like Lipase/Acylhydrolase | 8 |
| PF01083 | Cutinase | 8 |
| PF03443 | Glyco_hydro_61 | 8 |
| PF00135 | Carboxylestase family | 6 |
| PF00150 | Cellulase (glycosyl hydrolase family 5) | 6 |
| PF00264 | Common central domain of tyrosinase | 6 |
| PF01565 | FAD binding domain | 6 |
| PF04616 | Glycosyl hydrolases family 43 | 6 |
| PF00732 | GMC oxidoreductase | 5 |
| PF03211 | Pectate lyase | 5 |
| PF05730 | CFEM domain | 5 |
|  |  |  |
| Number: the total number of secreted protein in protein family | |  |
