# Supplementary material for: Sequencing the genome of Marssonina brunnea reveals fungus-poplar co-evolution
Source: BMC Genomics. 2012 Aug 9;13:382. doi: 10.1186/1471-2164-13-382 (PMC3484023; doi:10.1186/1471-2164-13-382)
Supplement: Additional file 19 — Table S12. The secretory protein families with more than five members M. brunnea. [file 1471-2164-13-382-S19.doc]

Table S12 Secretory protein families with more than five members in *M. brunnea*.

| Pfam ID | Family Name | Number |
| --- | --- | --- |
| PF01476 | LysM | 29 |
| PF00544 | Pec_lyase_C | 8 |
| PF00657 | GDSL-like Lipase/Acylhydrolase | 8 |
| PF01083 | Cutinase | 8 |
| PF03443 | Glyco_hydro_61 | 8 |
| PF00135 | Carboxylestase family | 6 |
| PF00150 | Cellulase (glycosyl hydrolase family 5) | 6 |
| PF00264 | Common central domain of tyrosinase | 6 |
| PF01565 | FAD binding domain | 6 |
| PF04616 | Glycosyl hydrolases family 43 | 6 |
| PF00732 | GMC oxidoreductase | 5 |
| PF03211 | Pectate lyase | 5 |
| PF05730 | CFEM domain | 5 |
|  |  |  |
| Number: the total number of secreted protein in protein family | |  |
