# Supplementary material for: Sequencing the genome of Marssonina brunnea reveals fungus-poplar co-evolution
Source: BMC Genomics. 2012 Aug 9;13:382. doi: 10.1186/1471-2164-13-382 (PMC3484023; doi:10.1186/1471-2164-13-382)
Supplement: Additional file 20 — Table S2. The GenBank accession no of ITS sequences used for phylogenetic tree analysis. [file 1471-2164-13-382-S20.doc]

Table S2 The GenBank accession no of ITS sequences used for phylogenetic tree analysis.

| Species | Strain | Accession no |
| --- | --- | --- |
| *Botrytis cinerea* | BC_1283 | EF207415 |
| *Botrytis cinerea* | BC_BC12 | GU724512 |
| *Botrytis cinerea* | BC_FSU6300 | GQ221113 |
| *Sclerotinia_sclerotiorum* | SS_ms82 | HQ833447 |
| *Sclerotinia_sclerotiorum* | SS_ms83 | HQ833448 |
| *Sclerotinia_sclerotiorum* | SS_ms84 | HQ833449 |
| *Marssonina coronariae* | MC_KR-AP-1 | GQ456166 |
| *Marssonina coronariae* | MC_LLH-M8-12 | HM368521 |
| *Marssonina coronariae* | MC_LLHs-196 | HM368520 |
| *Marssonina coronariae* | MC_ZXR-YL-Ye-1 | EU329735 |
| *Marssonina coronariae* | MC_ZXR-YL-Ye-2 | EU329734 |
| *Marssonina coronariae* | MC_ZXR-YL-Ye-3 | EU329732 |
| *Marssonina coronariae* | MC_ZXR-bshb-1 | EU329733 |
| *Marssonina coronariae* | MC_lk3 | FJ606802 |
| *Marssonina coronariae* | MC_lsl | FJ606800 |
| *Marssonina coronariae* | MC_wg5l | FJ606799 |
| *Marssonina coronariae* | MC_wgl | FJ606798 |
| *Marssonina coronariae* | MC_zgh | FJ606801 |
| *Marssonina rosae* | MR_26L-435-Mexico | AY904059 |
| *Marssonina rosae* | MR_RM071209004 | FJ493247 |
| *Marssonina rosae* | MR_RM071209006 | FJ493242 |
| *Marssonina rosae* | MR_RM080715007 | FJ493248 |
| *Marssonina rosae* | MR_RM080715008 | FJ493249 |
| *Marssonina rosae* | MR_UASWS0428 | HM235978 |
